# Dynamic Control of Chiral Recognition in Water-Soluble Naphthotubes Induced by Hydrostatic Pressure

**DOI:** 10.1021/acsnanoscienceau.4c00052

**Published:** 2024-10-21

**Authors:** Junnosuke Motoori, Tomokazu Kinoshita, Hongxin Chai, Ming-Shuang Li, Song-Meng Wang, Wei Jiang, Gaku Fukuhara

**Affiliations:** † Department of Chemistry, Tokyo Institute of Technology, 2-12-1 Ookayama, Meguro-ku, Tokyo 152-8551, Japan; ‡ Shenzhen Xinhua Middle School, Shenzhen 518109, China; § Department of Chemistry, Southern University of Science and Technology, Shenzhen 518055, China

**Keywords:** chiral naphthotube, hydrostatic pressure, chiral recognition, enantioselectivity, dynamic control

## Abstract

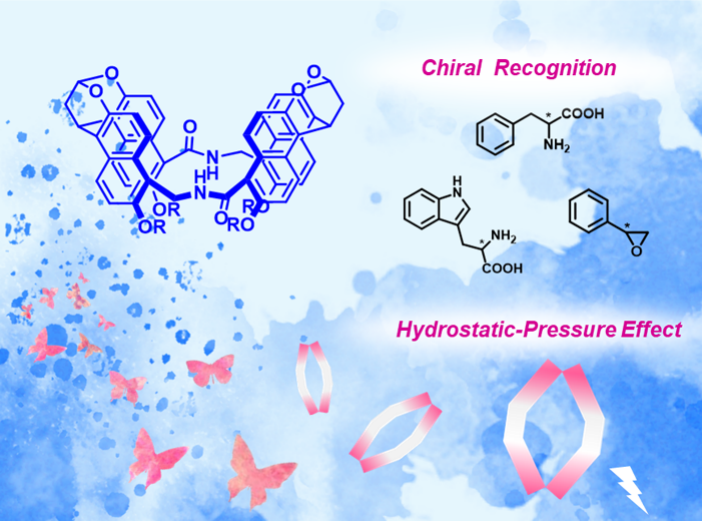

The dynamic control of chiral (enantiomeric) responses in chiral host–guest complexes through external stimuli is a significant challenge in modern chemistry for developing smart stimuli-responsive materials. Herein, we report the (chir)optical properties and chiral recognition behavior of water-soluble chiral naphthotubes (**1**) under the influence of hydrostatic pressure as an external stimulus. The hydrostatic pressure spectral profiles compared to those obtained at normal pressure revealed the dynamic behavior of **1** under hydrostatic pressure, owing to the flexible linker. In chiral recognition experiments, hydrophilic amino acids such as phenylalanine (Phe) and tryptophan (Trp) exhibited reaction volume changes (Δ*V*°) of −0.9 cm^3^ mol^–1^ for d-Phe, −1.2 cm^3^ mol^–1^ for l-Phe, −5.6 cm^3^ mol^–1^ for d-Trp, and −7.0 cm^3^ mol^–1^ for l-Trp, with enantioselectivity ranging from 1.2 to 1.6. In contrast, hydrophobic chiral styrene oxide (**2**) showed Δ*V*° values of 1.5 cm^3^ mol^–1^ for *R*-**2** and 3.5 cm^3^ mol^–1^ for *S-***2**, with a relatively higher enantioselectivity of up to 7.6. These contrasting effects of hydrostatic pressure primarily originate from the dynamics of chiral naphthotubes.

## 1 Introduction

The development of smart host molecules, such as artificial receptors and synthetic chemosensors, has garnered significant attention in multidisciplinary chemistry, particularly in the field of supramolecular chemistry.^1−6^ Such smart supramolecular materials are promising candidates for potential applications in molecular memories, logic gates, and drug delivery systems.^7−9^ In particular, enantiomeric responses induced by chiral guest molecule complexation are unique characteristics that can be harnessed in molecule-based devices such as 3D optical displays, security tags, and so on.^10−13^ Currently, the focus is shifting toward controlling such chiral recognition responses, which is expected to lead to the development of even smarter supramolecular systems.^14−16^ Therefore, in order to dynamically control chiral recognition in supramolecular complexation, a wide variety of external stimuli—such as solvent,^17−19^ temperature,^20,21^ electronic excitation,^22,23^ and mechanical forces^24−26^ (stress, strain, and pressure)—have been applied.

Recently, hydrostatic pressure or solution-state isotropic pressure has regained attention despite being an old topic, since many aspects, functions, and concepts in “mechano”-science are continually being discovered.^25^ Nevertheless, mysteries remain in mechanochemical^27−29^ and mechanobiological systems,^30−32^ i.e., how, to what extent, and where hydrostatic pressure stimuli affect these targets. Here, we exclude high-pressure solid chemistry using a diamond anvil cell at approximately GPa,^33,34^ which is beyond our target range of ca. MPa under hydrostatic pressure. Hydrostatic pressure effects in solution media have been investigated since the 1960s,^35−45^ wherein some host–guest supramolecular systems under hydrostatic pressure have been examined.^46−53^ However, so far, the hydrostatic pressure stimulus on chiral recognition upon supramolecular complexation has been little regarded as such a dynamic control effector. Very recently, we reported hydrostatic pressure-induced chiral responses upon the complexation of a chiral ion pair (guest) and a fluorescent anion receptor (host) with relatively effective reaction volume changes (Δ*V*°) of 2.3–9.7 cm^3^ mol^–1^.^54^ Hence, this finding encouraged us to newly explore an appreciable host-chiral guest combination that can be dynamically controlled by hydrostatic pressure. The good explanation of Δ*V*° in value and sign (instead of Δ*G*°) was illustrated in the previous host–guest system.^25,47^

**Figure 1:**
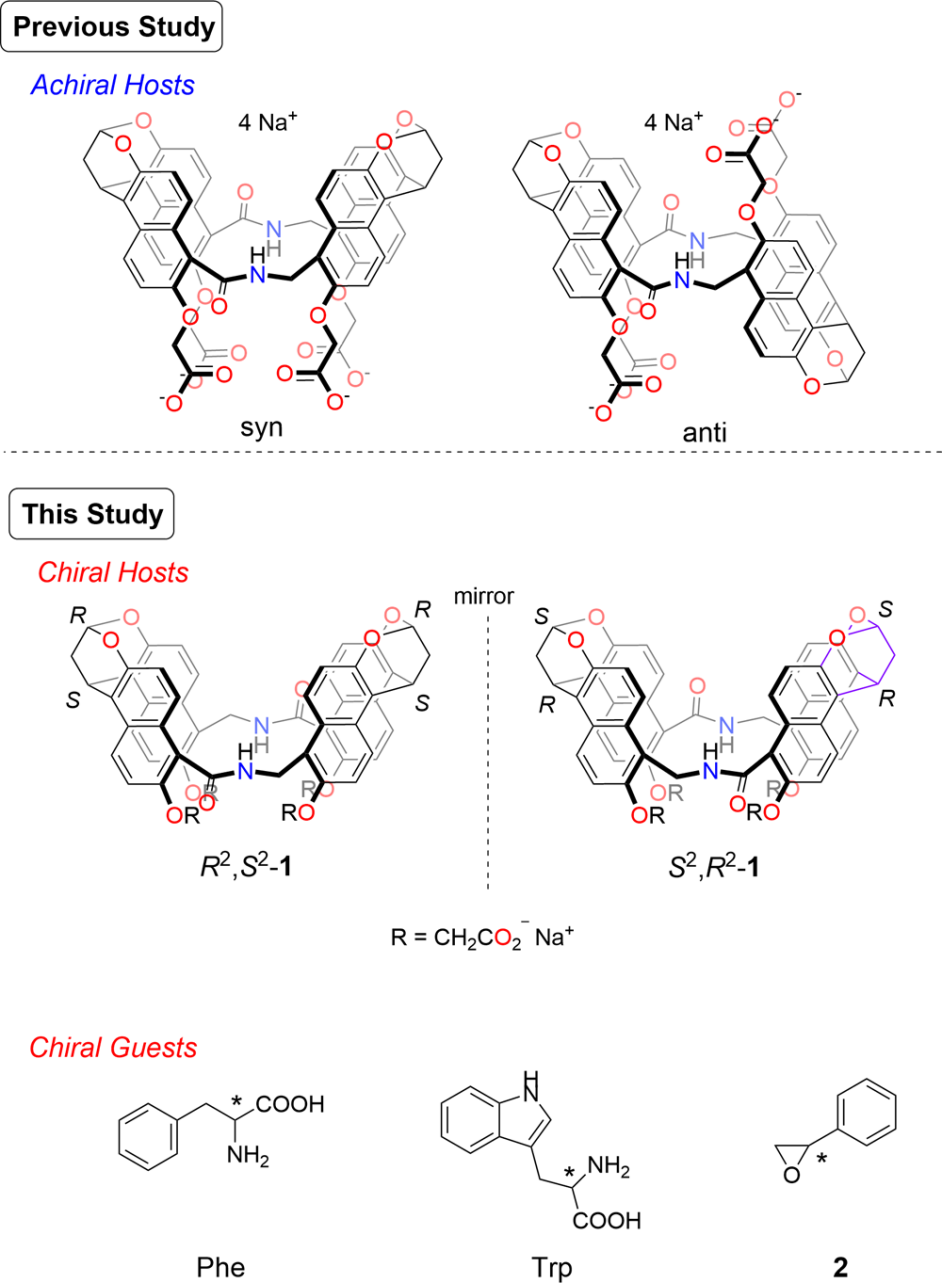
Chemical structures: achiral naphthotubes (top), chiral naphthotubes, and chiral guests used in this study (bottom).

In this study, to dynamically control chiral responses stimulated by hydrostatic pressure, we focused on a chiral water-soluble naphthotube.^55,56^ Naphthotubes are smart host molecules wherein naphthalene walls are connected by a flexible methylene linker with polar functional groups.^56^ Therefore, chiral naphthotubes (*R*^2^,*S*^2^-, and *S*^2^,*R*^2^-**1**, Figure 1 (bottom))^55^ provide a deeper hydrophobic cavity and a polar binding site, the combination of which plays an important role in the chiral discrimination ability in H_2_O. This cooperative binding motif differs from that operative in other water-soluble chiral hosts, e.g., cyclodextrins,^57,58^ chirally modified calixarenes,^59^ -cucurbiturils,^60^ -pillararenes,^61^ and other molecular receptors.^62−66^ Indeed, at an ambient pressure (0.1 MPa), the chiral naphthotube showed relatively good enantioselectivities (*K*_S_/*K*_R_ or *K*_R_/*K*_S_) of up to 2.0 in H_2_O when using a series of chiral styrene oxide guests.^55^ More importantly, as shown in Figure 1 (top), achiral naphthotube derivatives (*anti*- and *syn*-isomers) exhibited good hydrostatic pressure effects on Δ*V*° as −6.3 (to *anti*) and 3.2 cm^3^ mol^–1^ (to *syn*) for 1,4-dioxane.^67^ These previous findings may provide us with a hint that the chiral naphthotube will function as an excellent candidate toward a pressure-responsive smart chiroptical material induced by chiral molecule complexation. Herein, we report the dynamic control of the chiral naphthotube (*S*^2^,*R*^2^-**1**) during chiral recognition induced by hydrostatic pressurization. For this purpose, we chose the enantiomeric pairs of phenylalanine (d/l-Phe), tryptophan (d/l-Trp), and styrene oxide (**2**) as hydrophilic guests for the former two and hydrophobic guests for the latter. The results obtained herein provide deeper insights into the factors governing the hydrostatic pressure effect of chiral naphthotubes.

## 2 Materials and Methods

### 2.1 Materials

All commercial reagents were used as received without further purification. Fluorescence-free grade water (Milli-Q) was used for spectroscopy. Chiral naphthotubes (*R*^2^,*S*^2^-, and *S*^2^,*R*^2^-**1**) were synthesized according to a literature procedure.^55^

### 2.2 Instruments

The UV/vis spectra were measured by using a JASCO V-650 spectrometer. Fluorescence spectra were measured by using a JASCO FP-8500 instrument. The fluorescence lifetime decay profiles were obtained by using a Hamamatsu Quantaurus-Tau single-photon counting apparatus fitted with an LED light source. Circular dichroism (CD) spectra were obtained by using a JASCO J-720WI instrument.

### 2.3 Hydrostatic Pressure Spectroscopy

Spectroscopic experiments under hydrostatic pressures were conducted using a custom-built high-pressure apparatus; the details are summarized in our previous study.^25^ Concisely, a quartz inner cell (2 mm path length) with a Teflon tube was filled with an H_2_O solution of the sample. The cell was then placed into the outer cell, wherein sapphire windows were fitted. The tightly packed outer cell was placed in the spectrometers and hydrostatically pressurized in the range of 0.1–400 MPa. The photographs are shown in Figure S1 in the Supporting Information (SI).^25,68^

## 3 Results and Discussion

### 3.1 (Chir)optical Properties and Molecular Recognition Behavior of 1

**Figure 2:**
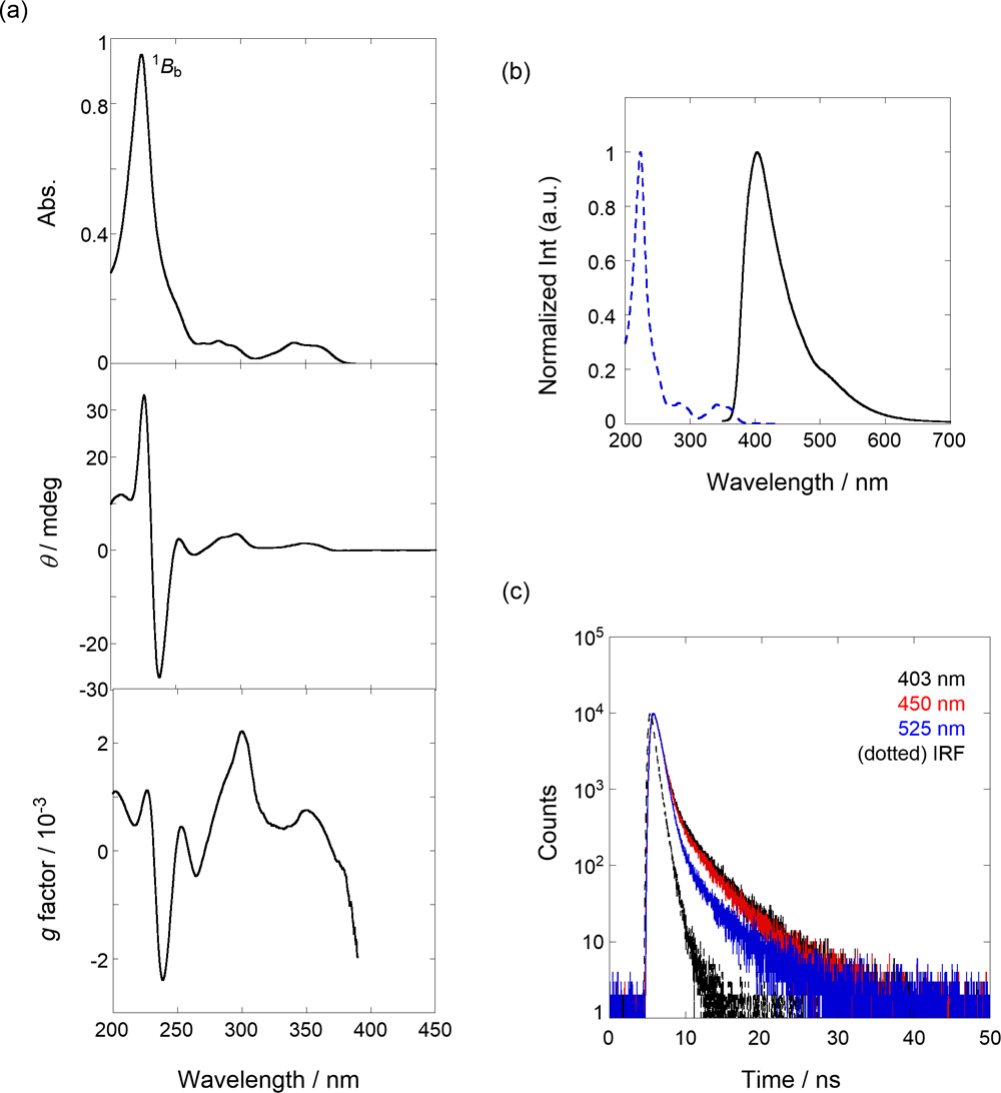
(a) UV/vis (top), CD (middle), and *g* factor spectra (bottom) of **1** (9.01 μM) in H_2_O at 25 °C. (b) Fluorescence spectrum (λ_ex_: 300 nm, black solid line) of **1** (9.01 μM) in H_2_O at 25 °C; the blue dotted line represents the normalized UV/vis spectrum. (c) Fluorescence lifetime decays (λ_ex_ 280 nm) of **1** (9.01 μM), monitored at 403 (black), 450 (red), and 525 nm (blue) at room temperature. All spectra were measured in a 1 cm cell.

Before subjecting the compound to hydrostatic pressurization experiments, we investigated the (chir)optical properties and molecular recognition behavior of **1** in H_2_O at 0.1 MPa. Although we previously measured circular dichroism (CD) spectra,^55^ we did not focus on the main band in detail. Here, we report the detailed chiroptical properties of **1**. As shown in Figure 2a, at the main band based on the long axis (^1^*B*_b_ transition) of the naphthalene chromophore, a strong bisignate couplet was observed in the anisotropy (*g*) factor profiles; *g*_239 nm_ = −0.0024, *g*_226 nm_ = 0.0011. According to the exciton chirality theory,^69^ the observed negative exciton coupling suggests that the four naphthalene walls in *S*^2^,*R*^2^-**1** were aligned in a left-handed manner. In the fluorescence spectra (Figure 2b), a slightly large Stokes shift of 3280 cm^–1^ was observed despite the naphthalene chromophore, indicating excited-state flexibility or relaxation in the chiral naphthotube. As shown in Figure 2c, fluorescence lifetime decays (λ_em_: 403, 450, and 525 nm) were reasonably fitted to a sum of two exponential functions to afford τ_1_ as 0.4 and τ_2_ as 3.5 ns, respectively, as listed in Table 1; all decay fitting data are shown in Figures S2–S4 in SI. By shifting the observed wavelength from 403 to 525 nm, τ_1_ species was preferred (*A*_1_: 0.81–0.94), thus indicating that the short-lived species are located in the longer wavelength region; in contrast to τ_2_ at the shorter wavelength. Therefore, the longer-lived species τ_2_ was ascribed to monomer-state naphthalene. In addition, the short-lived τ_1_ can be assigned to the intramolecular ground-state stacked species, according to the promoted radiationless deactivation process.^70^ In particular, based on the structural features observed in the X-ray single crystal,^55^ this may occur in the bis(naphthalene) cleft connected by the flexible cycloalkoxy group (purple moiety in Figure 1a (bottom)); the packing structure was given in the previous report.^55^ The fluorescence quantum yield (Φ_F_) was 0.07, for which the major stacked species in H_2_O is highly likely responsible.

**Table 1:** Fluorescence Lifetimes of 1 in the Absence and Presence of 1,4-Dioxane in H_2_O at Room Temperature^a^

compd.	λ_em_^b^ (nm)	τ_1_ (ns)	*A*_1_	τ_2_ (ns)	*A*_2_	χ^2^
**1**^c^	403	0.4	0.81	3.5	0.19	1.3
	450	0.4	0.85	3.5	0.15	1.2
	525	0.4	0.94	3.5	0.06	1.1
**1** + 1,4-dioxane^d^	403	1.6	1.00			1.3
	450	1.6^e^	0.99	3.6	0.01	1.3
	525	1.6	0.95	3.9	0.05	1.3

a Fluorescence lifetime (τ_i_) and relative abundance (*A*_i_) of each excited species, determined by the single-photon counting method in nondegassed solution.

b Monitoring wavelength.

c [**1**] = 9.01 μM.

d [**1**] = 9.63 μM, [1,4-dioxane] = 5.56 mM.

e Fixed.

### 3.2 Complexation-Induced Optical Properties of 1: Dynamic Flapping

**Figure 3:**
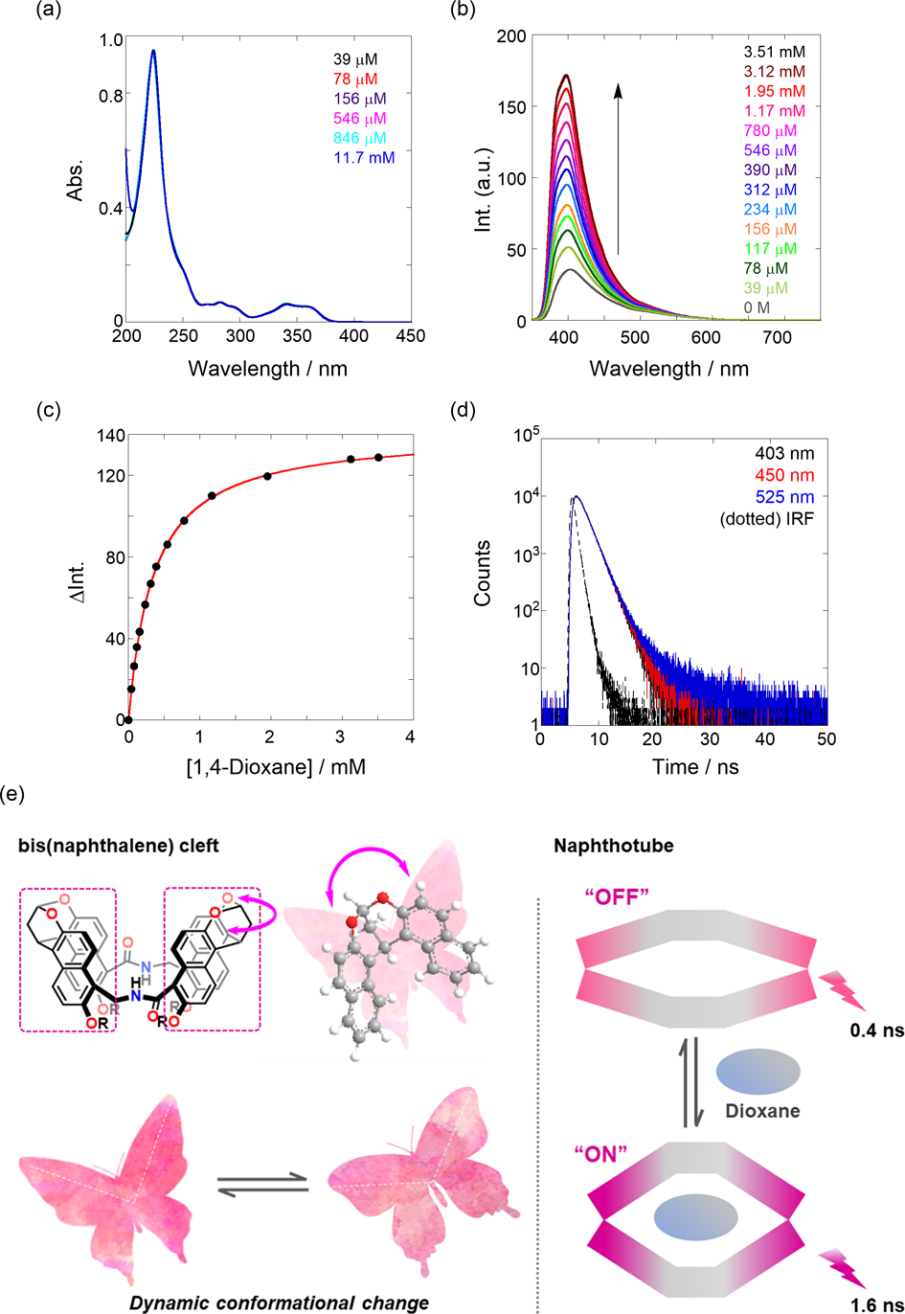
(a) UV/vis and (b) fluorescence spectra (λ_ex_ 300 nm) of **1** (9.01 μM) upon the addition of 1,4-dioxane (0–3.51 mM, colored lines) in H_2_O at 25 °C. (c) Nonlinear least-squares fitting, assuming a 1:1 stoichiometry, to determine the binding constant (*K*) of 1,4-dioxane with **1**. (d) Fluorescence lifetime decays (λ_ex_ = 280 nm) of **1** (9.63 μM) and 1,4-dioxane (5.56 mM), monitored at 403 (black), 450 (red), and 525 nm (blue) at room temperature. All spectra were measured in a 1 cm cell. (e) Schematic illustration of the (*left*) dynamic flapping and (*right*) turn-on mechanism of **1**.

Second, we investigated the optical properties affected by the complexation of a guest molecule at 0.1 MPa. As shown in Figure 3a,b, the gradual addition of 1,4-dioxane, which was used as a guest molecule for achiral naphthotubes with a binding constant (*K*) of 10^3^–10^4^ M^–1^,^67^ caused a steady increase in the fluorescence intensity, despite negligible changes in the UV/vis spectra. According to the previous binding stoichiometry,^67^Figure 3c shows the nonlinear least-squares fitting of the fluorescence titration data, assuming a 1:1 stoichiometry, which afforded *K* as 2930 ± 30 M^–1^, comparable to that obtained in the achiral naphthotube. To gain deeper mechanistic insights, we measured the fluorescence lifetime decay, as shown in Figure 3d. The decay profiles with and without 1,4-dioxane were also reasonably fitted to two exponentials of τ_1_ as 1.6 and τ_2_ as 3.6–3.9 ns (the fitting data are shown in Figures S2 and S3 in SI). The extended τ_1_ (0.4 → 1.6 ns) can be applicably accounted in terms that the intramolecular stack in the bis(naphthalene) cleft was canceled out due to the inclusion of the bulky guest. The plausible dynamic behavior of compound **1** is illustrated in Figure 3e. If the cleft works as a hinge, **1** is most likely to induce butterfly-like flapping upon guest addition. Eventually, this caused the gradual *turn-on* of fluorescence signals upon the stepwise addition of hydrophobic guests. At a host occupancy >99.9%, based on the addition of 1,4-dioxane (3.51 mM) to an H_2_O solution of **1** (9.01 μM), the Φ_F_ value improved to 0.20, supporting the turn-on mechanism.

### 3.3 Hydrostatic Pressure Effects on 1

**Figure 4:**
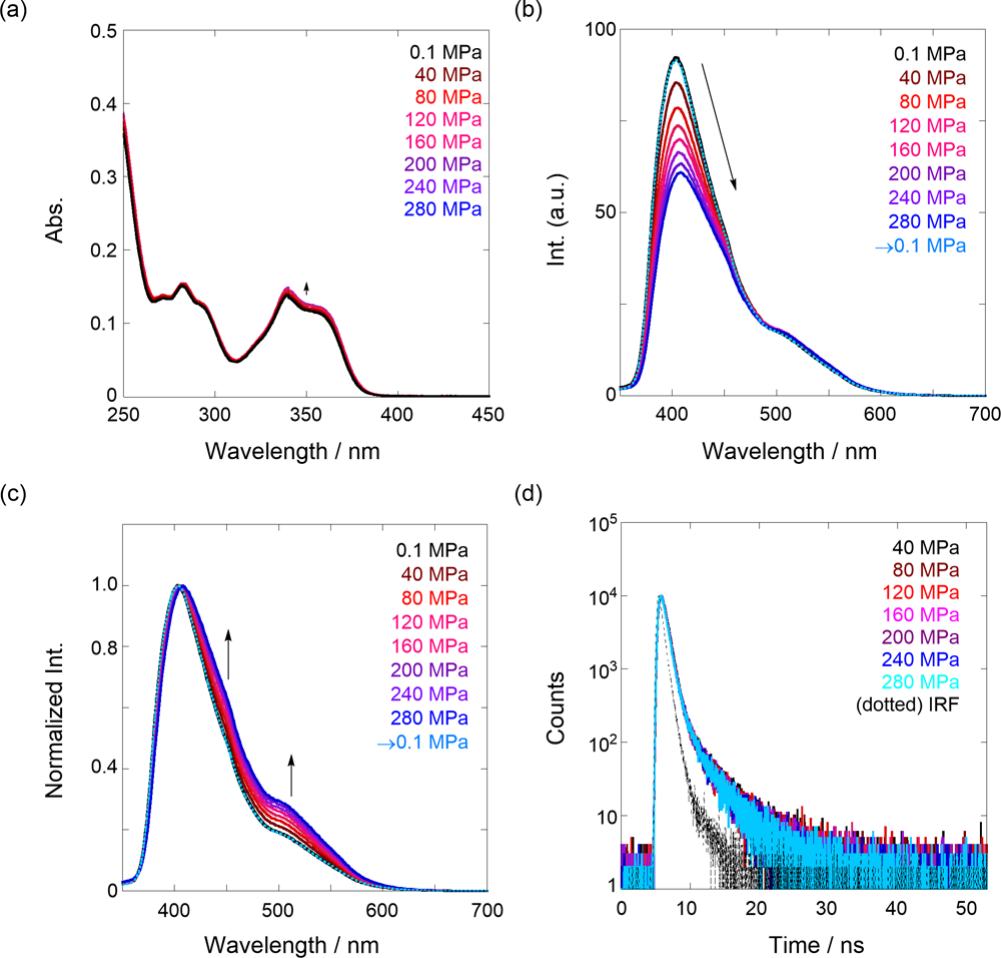
Pressure-dependent UV/vis (a) (90.1 μM), fluorescence (b) (9.63 μM, λ_ex_ 300 nm), normalized fluorescence spectra (c), and fluorescence lifetime decays (d) (87.9 μM, λ_ex_ 280 nm, λ_em_ 525 nm) of **1** in H_2_O at room temperature over the range of 0.1–280 MPa. All spectra were measured in a high-pressure cell.

Next, we measured the hydrostatic pressure spectroscopy of **1** in the absence of chiral guests. As shown in Figure 4a, a gradual increase in absorbance upon hydrostatic pressurization was observed simply because of the increase in the effective concentration by pressurization. Interestingly, in Figure 4b, the fluorescence intensities gradually decreased, although in general, the intensity of fluorophores increased upon hydrostatic pressurization owing to the inhibition of solvent attack in the excited state, based on the increasing viscosity of the solution used.^45^ This contrasting fluorescence behavior in **1** vs other fluorophores is well-understood by normalizing the fluorescence spectra (Figure 4c), indicating an increasing amount of intramolecular stacked species in the longer wavelength region. To confirm this further, the hydrostatic pressure lifetime decay (Figure 4d) was measured. As listed in Table 2 and Figure S4, the short-lived τ_1_ was further decreased from 0.42 to 0.35 ns with increasing hydrostatic pressure, indicating that the intramolecular stacking was further promoted (more stacked). The long-lived τ_2_ was also decreased from 3.2 to 2.3 ns with elevating pressure, which may be originated from gradual deactivation by pressure-induced solvent attack in the naphthalene chromophore moiety (see Figure 6c, left). Again, this suggests that **1**, particularly at the bis(naphthalene) cleft, is flexible or *dynamic* upon hydrostatic pressurization. In addition, the depressurized fluorescence spectrum (0.1 from 280 MPa (Figure 4b, sky blue line)) was superimposable on the original spectrum of 0.1 MPa (Figure 4b, black line), indicating that dynamic stacking is a reversible process.

**Table 2:** Fluorescence Lifetimes of 1 upon the Hydrostatic Pressurization in H_2_O at Room Temperature^a^

pressure (MPa)	τ_1_ (ns)	*A*_1_	τ_2_ (ns)	*A*_2_	χ^2^
40	0.42	0.93	3.2	0.07	1.1
80	0.40	0.92	3.0	0.08	1.0
120	0.39	0.92	2.8	0.08	1.2
160	0.38	0.92	2.9	0.08	1.2
200	0.36	0.90	2.4	0.10	1.3
240	0.36	0.90	2.3	0.10	0.9
280	0.35	0.90	2.3	0.10	1.1

a Fluorescence lifetime (τ_i_) and relative abundance (*A*_i_) of each excited species, determined by the single-photon counting method in nondegassed solution; [**1**] = 87.9 μM, λ_em_ 525 nm.

### 3.4 Achiral Guest Complexation of 1 upon Hydrostatic Pressurization

For the hydrostatic pressure-binding behavior, the pressure effect of **1** upon complexation was first investigated by using achiral 1,4-dioxane. As shown in Figure S5, the gradual addition of 1,4-dioxane to an H_2_O solution of **1** at different pressures (40–200 MPa) caused a steady increase in fluorescence intensity. Therefore, this “turn-on” signaling based on the guest complexation under hydrostatic pressures is most originated from the dynamic flexibility of the bis(naphthalene) cleft (stacking on/off), which is supported by the ambient and hydrostatic pressure fluorescence lifetime measurements (vide supra). Similar to the *K* value obtained at 0.1 MPa, nonlinear least-squares fitting of the fluorescence increase at each pressure yielded *K* values, as listed in Table 3. To further evaluate the hydrostatic pressure effect of **1** upon complexation more quantitatively, we calculated Δ*V*° according to eq 1:
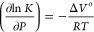
1

**Table 3:** Binding Constants (*K*), Enantioselectivity, and Reaction Volume Changes (Δ*V*°) for 1:1 Complexation of Achiral and Chiral Guests with 1 in H_2_O under Hydrostatic Pressure at Room Temperature^a^

guest	pressure (MPa)	*K*^b^ (M^–1^)	*K*_L_/*K*_D_	Δ*V*° (cm^3^ mol^–1^)
			*K*_S_/*K*_R_	
1,4-dioxane	0.1	2902 ± 245	c	1.2 ± 0.3
	40	2924 ± 213		
	80	2739 ± 230		
	120	2699 ± 237		
	160	2662 ± 241		
	200	2675 ± 267		
l-Phe	40	260 ± 20	1.5	–1.2 ± 0.2
	80	253 ± 21	1.3	
	120	264 ± 15	1.4	
	160	271 ± 21	1.5	
	200	277 ± 23	1.6	
	240	276 ± 25	1.3	
	280	289 ± 25	1.5	
d-Phe	40	178 ± 15	c	–0.9 ± 0.6
	80	189 ± 19		
	120	191 ± 21		
	160	186 ± 16		
	200	178 ± 22		
	240	210 ± 26		
	280	193 ± 30		
l-Trp	40	1241 ± 141	1.2	–7.0 ± 0.3
	80	1364 ± 115	1.2	
	120	1610 ± 119	1.2	
	160	1828 ± 146	1.3	
	200	1943 ± 94	1.2	
	240	2189 ± 168	1.3	
	280	2435 ± 180	1.4	
d-Trp	40	1016 ± 223	c	–5.6 ± 0.5
	80	1164 ± 266		
	120	1367 ± 281		
	160	1428 ± 293		
	200	1568 ± 327		
	240	1637 ± 344		
	280	1795 ± 370		
*S*-**2**	40	12877 ± 991	6.8	3.5 ± 0.5
	80	13618 ± 1142	7.6	
	120	12168 ± 944	6.7	
	160	12044 ± 1256	7.2	
	200	10878 ± 1541	6.4	
	240	10159 ± 1555	6.2	
	280	9649 ± 1553	5.9	
*R*-**2**	40	1903 ± 348	c	1.5 ± 0.3
	80	1786 ± 315		
	120	1812 ± 330		
	160	1673 ± 259		
	200	1689 ± 365		
	240	1648 ± 368		
	280	1648 ± 424		

a All titration experiments were performed in a high-pressure cell.

b For convenience, we reported values to the nearest whole number for the ln*K*-*P* plots.

c Not applicable.

**Figure 5:**
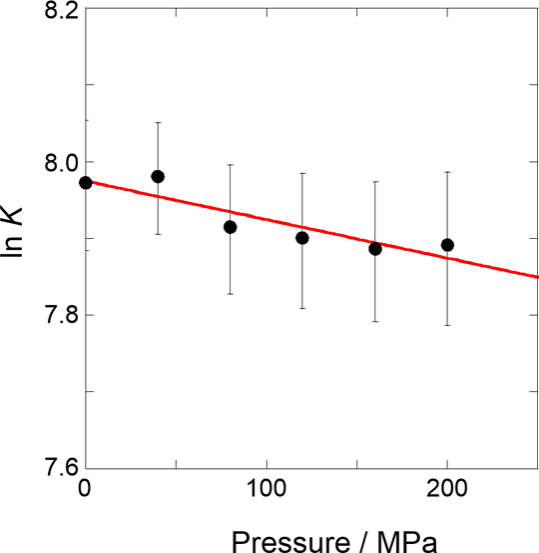
Pressure dependence of the binding constant (*K*) upon the complexation of 1,4-dioxane with **1** in H_2_O under hydrostatic pressures (0.1–200 MPa) at room temperature (correlation coefficient *r* = 0.903).

As shown in Figure 5, the natural logarithm of each *K* value obtained for 1,4-dioxane was plotted against pressure with a good linear relationship (*r* = 0.903), indicating that a single mechanism operated in the range of pressures studied. The Δ*V*° value obtained from the slope in the plot was 1.2 ± 0.3 cm^3^ mol^–1^, which is relatively small but positive. This is highly likely preferable for the tighter stacking of flexible naphthalene walls rather than the complexation-induced extension of the walls, resulting in a slight inhibition of supramolecular complexation upon hydrostatic pressurization. Therefore, the value and sign of Δ*V*° can provide us with the degree of dynamism in the naphthotube skeleton.

### 3.5 Amino Acids Complexation of 1 upon Hydrostatic Pressurization

**Figure 6:**
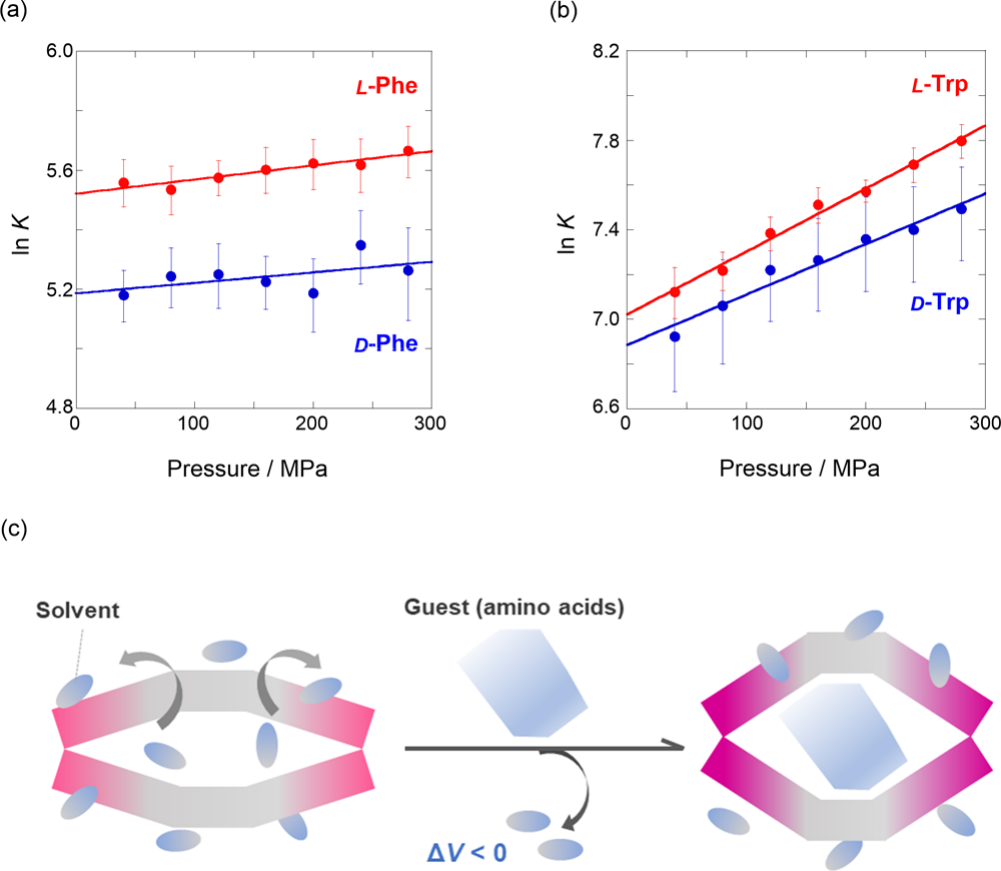
Pressure dependence of the binding constant (*K*) upon the complexation of (a) Phe (*r* = 0.541 for d, *r* = 0.938 for l) and (b) Trp (*r* = 0.981 for d, *r* = 0.995 for l) with **1** in H_2_O in a hydrostatic pressure range of 40–280 MPa at room temperature. (c) Schematic illustration of the dynamic stretch of **1** stimulated by guest inclusion upon hydrostatic pressurization.

Next, we investigated the hydrostatic pressure effects on **1** by using chiral guests. First, hydrophilic enantiomeric pairs (D/L) of Phe and Trp were investigated in a similar manner. In Figures S6 and S7, a similar trend for the complexation of Phe is observed, enabling the formation of *K* at each pressure (see Table 3). The enantioselectivity (*K*_L_/*K*_D_) of Phe ranged from 1.3 to 1.6, suggesting that the diastereomeric energy difference (**1** ⊂ d or l-Phe) had little effect on hydrostatic pressure stimulation. Interestingly, the obtained Δ*V*° values in the ln*K*-*P* plot (Figure 6a) were −0.9 ± 0.6 for d and −1.2 ± 0.2 cm^3^ mol^–1^ for l, which are relatively small and similar to those of 1,4-dioxane, but negative. This behavior can be reasonably explained by the preferential opening of the flexible naphthalene walls, causing a slight promotion of supramolecular complexation stimulated by hydrostatic pressure. This is most likely because the desolvation of H_2_O molecules around the hydrophilic functional groups (COO^–^ and NH_3_^+^) in Phe occurred upon complexation, leading to a decrease of Δ*V*° (negative sign), as illustrated in Figure 6c. A similar investigation of hydrophilic Trp provided stronger evidence of the dynamic behavior in naphthotubes. The routine hydrostatic pressure fluorescence titration of Trp (Figures S8 and S9) exhibited similar turn-on signaling to afford *K*_D_ and *K*_L_ at each pressure; the enantioselectivity varied in the range of 1.2–1.4 (see Table 3). More importantly, the ln*K*-*P* plot (Figure 6b) gave Δ*V*° as −5.6 ± 0.5 cm^3^ mol^–1^ for d and −7.0 ± 0.3 cm^3^ mol^–1^ for l, the larger value of which indicates the more dynamic open-up of the naphthalene walls upon hydrostatic pressurization. As is the case with Phe, this can be reasonably explained in terms of the much greater desolvation of hydrated H_2_O around the larger hydrophilic Trp moiety than that of Phe, which is most likely the origin.

### 3.6 Hydrophobic Chiral Guest Complexation of 1 upon Hydrostatic Pressurization: Toward Higher Enantioselectivity

**Figure 7:**
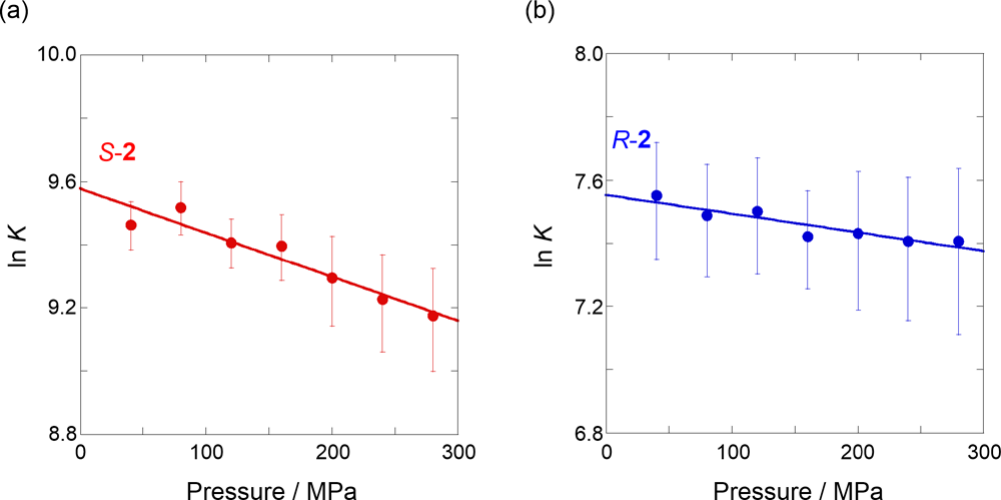
Pressure dependence of the binding constant (*K*) upon the complexation of (a) *S*-**2** (*r* = 0.955) and (b) *R*-**2** (*r* = 0.918) with **1** in H_2_O in a hydrostatic pressure range of 40–280 MPa at room temperature.

Finally, the extent to which the hydrophobic chiral guest (**2**) affected the dynamics of the naphthotube was investigated. A similar trend was observed when **2** was added to an H_2_O solution of **1** under high pressure (Figures S10 and S11, Table 3). Interestingly, the addition of the *S* enantiomer showed a larger turn-on signal in the lower concentration range than the *R* enantiomer, indicating stronger binding of the *S* enantiomer. Surprisingly, the enantioselectivity (*K*_S_/*K*_R_) varied in the range of 5.9–7.6, which is significantly higher than those obtained in other previous chiral hosts.^57−66^ This improved enantioselectivity, compared to that observed with hydrophilic amino acids, may be attributed to the dynamic naphthotube tightly conforming to the size and shape of the hydrophobic guest in an induced-fit manner. This was further supported by the Δ*V*° values obtained from the ln*K*-*P* plot: 3.5 ± 0.5 cm^3^ mol^–1^ for *S* (Figure 7a) and 1.5 ± 0.3 cm^3^ mol^–1^ for *R* (Figure 7b). Based on the characteristics of the dynamic naphthotube, the positive Δ*V*° can be easily explained by the strong solvation around hydrophobic guest **2**, which promotes the closing of the naphthalene walls. In other words, both **2** and the solvated H_2_O molecules strongly bind to the cavity in the naphthotubes, suggesting that such cosolvation plays a critical role in achieving higher chiral discrimination in a dynamic chiral host.

## 4 Conclusions

In conclusion, we demonstrated hydrostatic pressure-induced chiral recognition of both hydrophilic and hydrophobic guests using water-soluble chiral naphthotubes. The chiral naphthotube used in this study was relatively flexible and dynamic upon hydrostatic pressure stimulation due to its flexible linker. This dynamism critically determines the contrasting complexation of hydrophilic amino acids (negative Δ*V*°) and hydrophobic **2** (positive Δ*V*°), with the desolvation/solvation of water likely playing a significant role. Notably, a high enantioselectivity of up to 7.6 was achievable using a hydrostatic pressure control approach. Hence, this study provides valuable guidelines for the development of smart chiral chemosensors, materials, and devices.

## Supporting Information Available

Hydrostatic pressure apparatus, fluorescence lifetime decays, and titration data (DOCX)
