# Supplementary material for: Dynamic Control of Chiral Recognition in Water-Soluble Naphthotubes Induced by Hydrostatic Pressure
Source: ACS Nanosci Au. 2024 Oct 21;4(6):435–42. doi: 10.1021/acsnanoscienceau.4c00052 (PMC11659898; doi:10.1021/acsnanoscienceau.4c00052)
Supplement: Supplementary file 1 — ng4c00052_si_001.docx [file ng4c00052_si_001.docx]

***Supporting Information***

**for**

**Dynamic Control of Chiral Recognition in Water-Soluble Naphthotubes Induced by Hydrostatic Pressure**

Junnosuke Motoori,^†^ Tomokazu Kinoshita,^†^ Hongxin Chai,^‡^ Ming-Shuang Li,^§^ Song-Meng Wang,^§^ Wei Jiang,^§,¶^ and Gaku Fukuhara^*,†^

^†^ Department of Chemistry, Tokyo Institute of Technology, 2-12-1 Ookayama, Meguro-ku, Tokyo 152-8551, Japan

^‡^ Shenzhen Xinhua Middle School, Shenzhen 518109, China

^§^ Department of Chemistry, Southern University of Science and Technology, Shenzhen 518055, China

^¶^ This paper is dedicated to Prof. Wei Jiang, who passed away during this research.

Email: gaku@chem.titech.ac.jp

**Hydrostatic Pressure Apparatus**


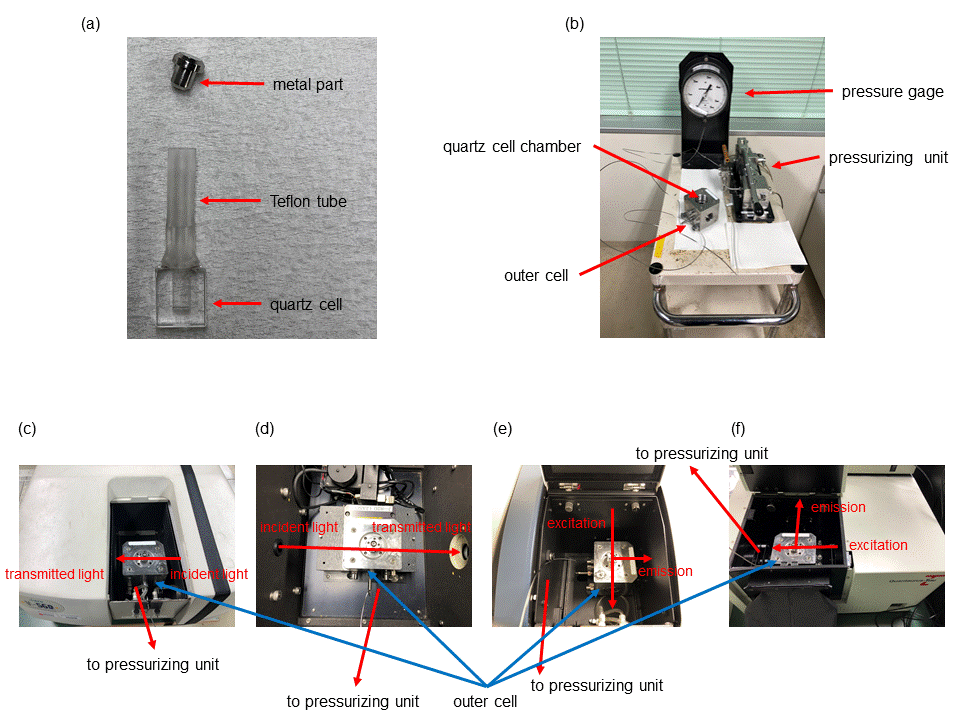


**Figure S1.** Photographs of (a) inner quartz cell, (b) pressurizing units, and setup for (c) UV/vis, (d) circular dichroism, (e) fluorescence, and (d) lifetime measurements. Reproduced with permission from ref 68. Copyright 2020 John Wiley & Sons.

**Fluorescence Lifetime Decays of 1**


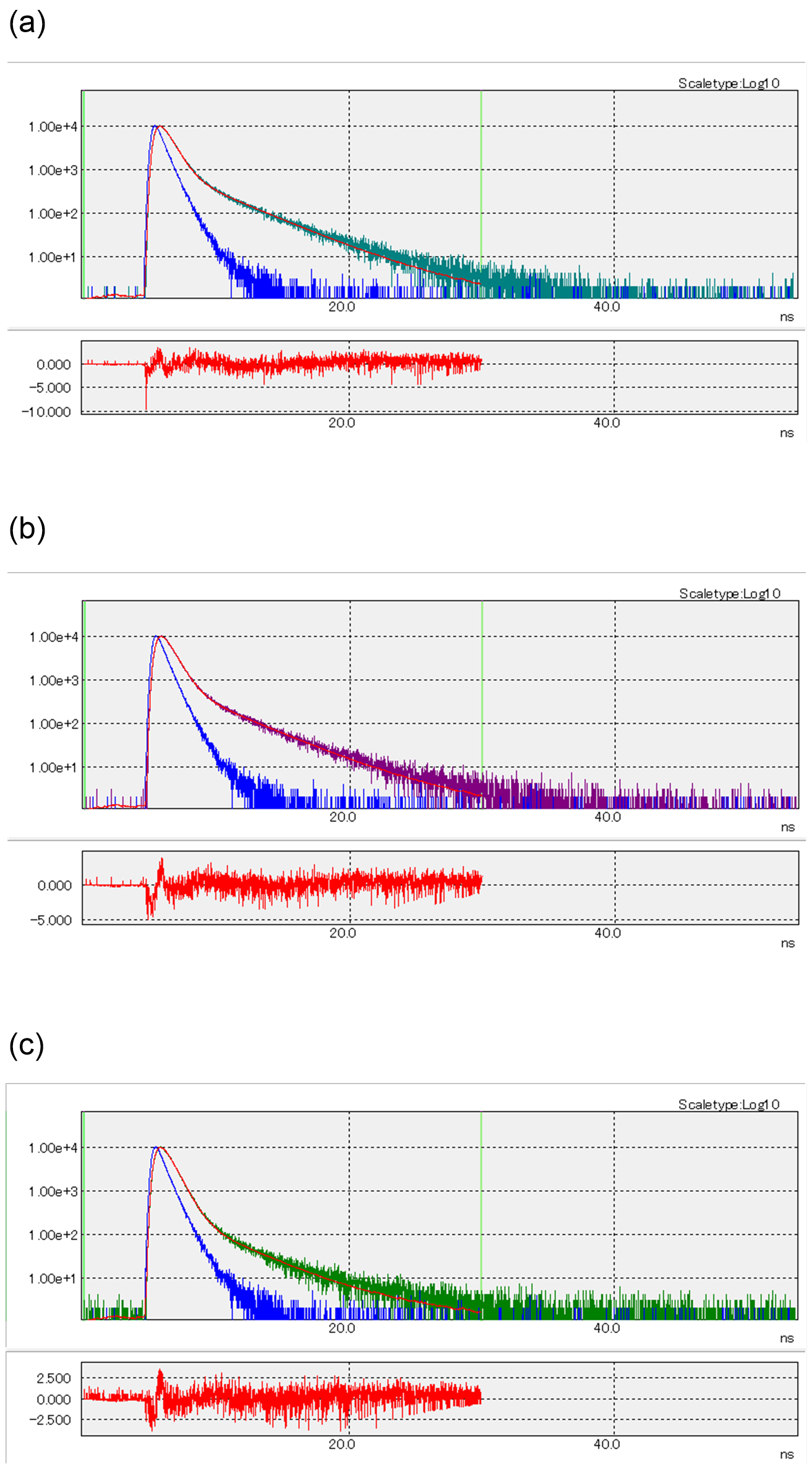


**Figure S2.** Time-correlated fluorescence decays of **1** (9.01 μM) monitored at (a) 403, (b) 450, and (c) 525 nm in H_2_O at room temperature, measured in a 1 cm cell, where the colored, red, and blue lines represent the fluorescence decay, fitting result, and the instrument response function, respectively.


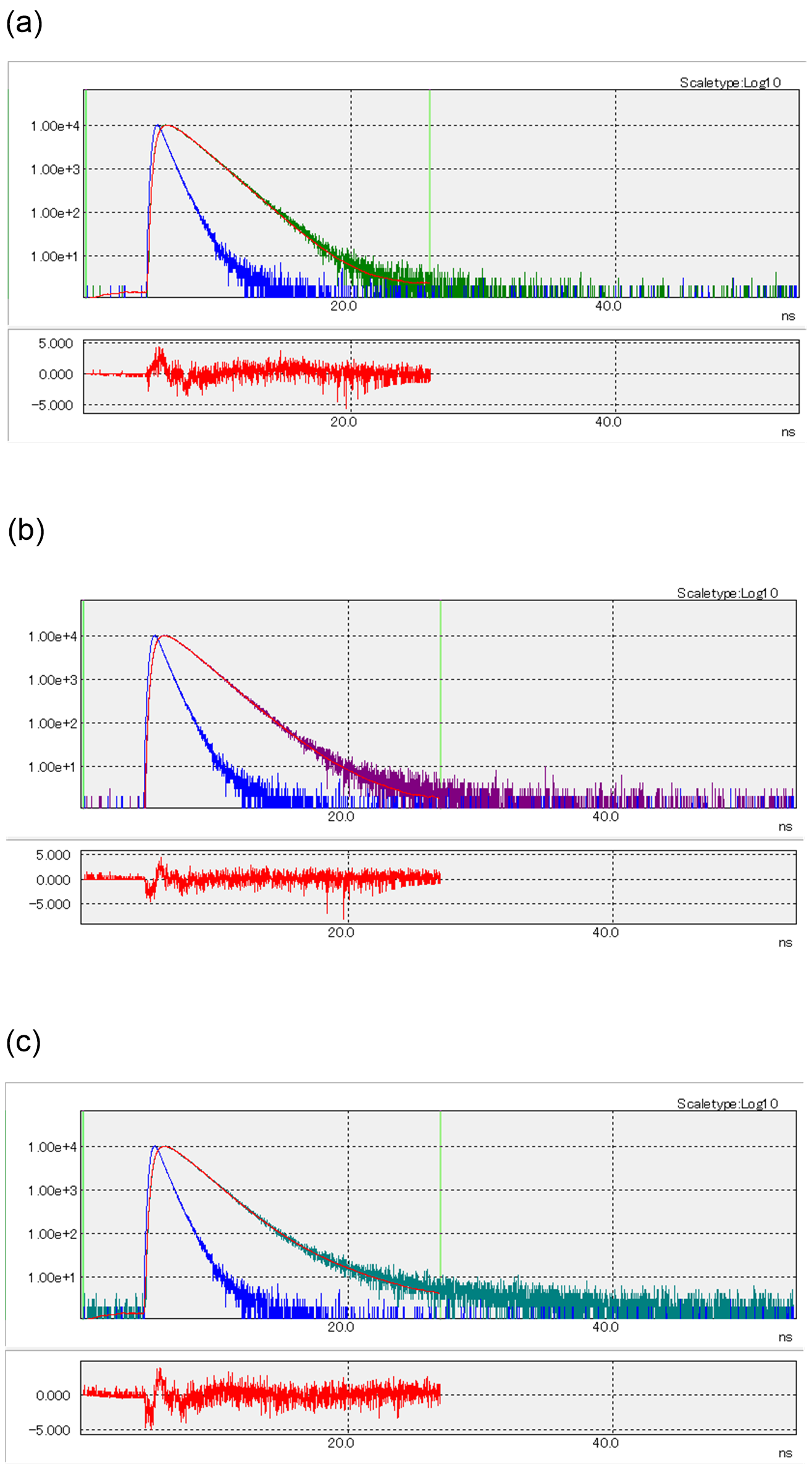


**Figure S3.** Time-correlated fluorescence decays of **1** (9.63 μM) upon the addition of 1,4-dioxane (5.56 mM) monitored at (a) 403, (b) 450, and (c) 525 nm in H_2_O at room temperature, measured in a 1 cm cell, where the colored, red, and blue lines represent the fluorescence decay, fitting result, and the instrument response function, respectively.


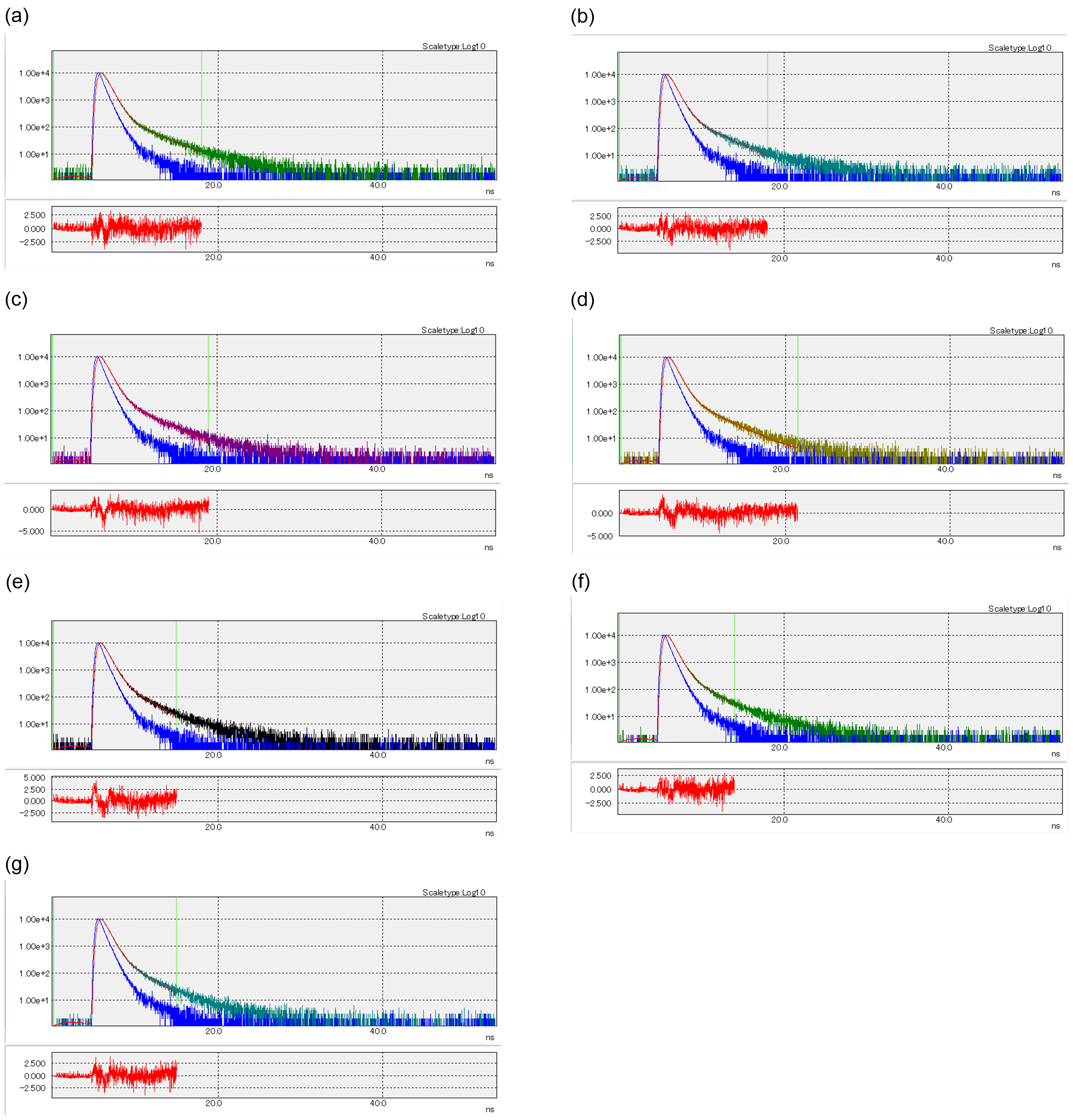


**Figure S4.** Time-correlated fluorescence decays of **1** (87.9 μM) monitored at 525 nm in H_2_O at (a) 40, (b) 80, (c) 120, (d) 160, (e) 200, (f) 240, and (g) 280 MPa at room temperature, measured in a high-pressure cell, where the colored, red, and blue lines represent the fluorescence decay, fitting result, and the instrument response function, respectively.

**Titration Data of 1**


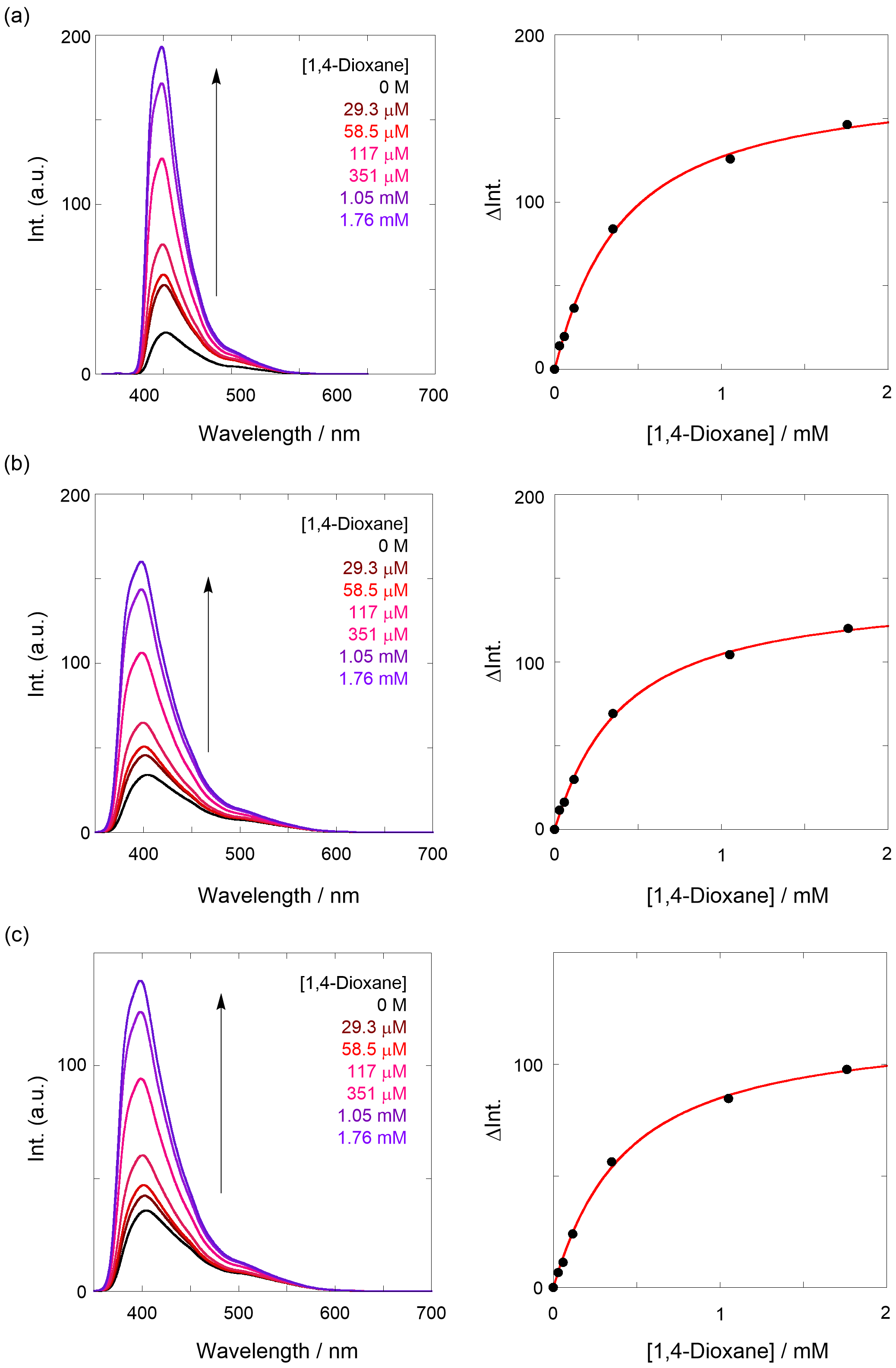


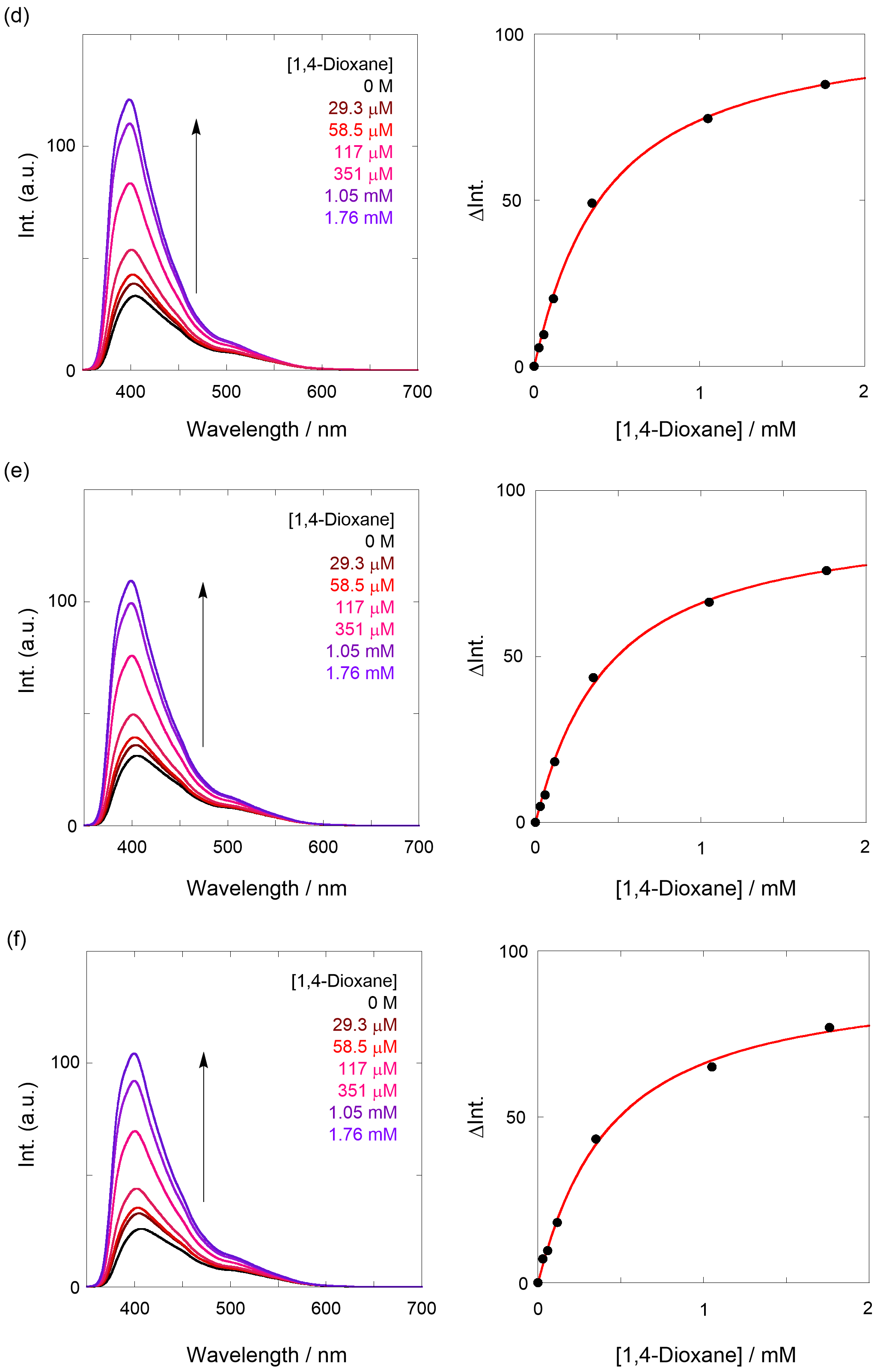


**Figure S5.** (*Left panels*) Fluorescence spectra (λ_ex_ 300 nm) of **1** (96.3 μM) upon the addition of 1,4-dioxane (0–1.76 mM, colored lines) in H_2_O at 25 °C, measured in a high-pressure cell. (*Right panels*) Nonlinear least-squares fitting, assuming a 1:1 stoichiometry, was used to determine the binding constant (*K*) of 1,4-dioxane to **1**. Pressure applied: (a) 0.1, (b) 40, (c) 80, (d) 120, (e) 160, and (f) 200 MPa.


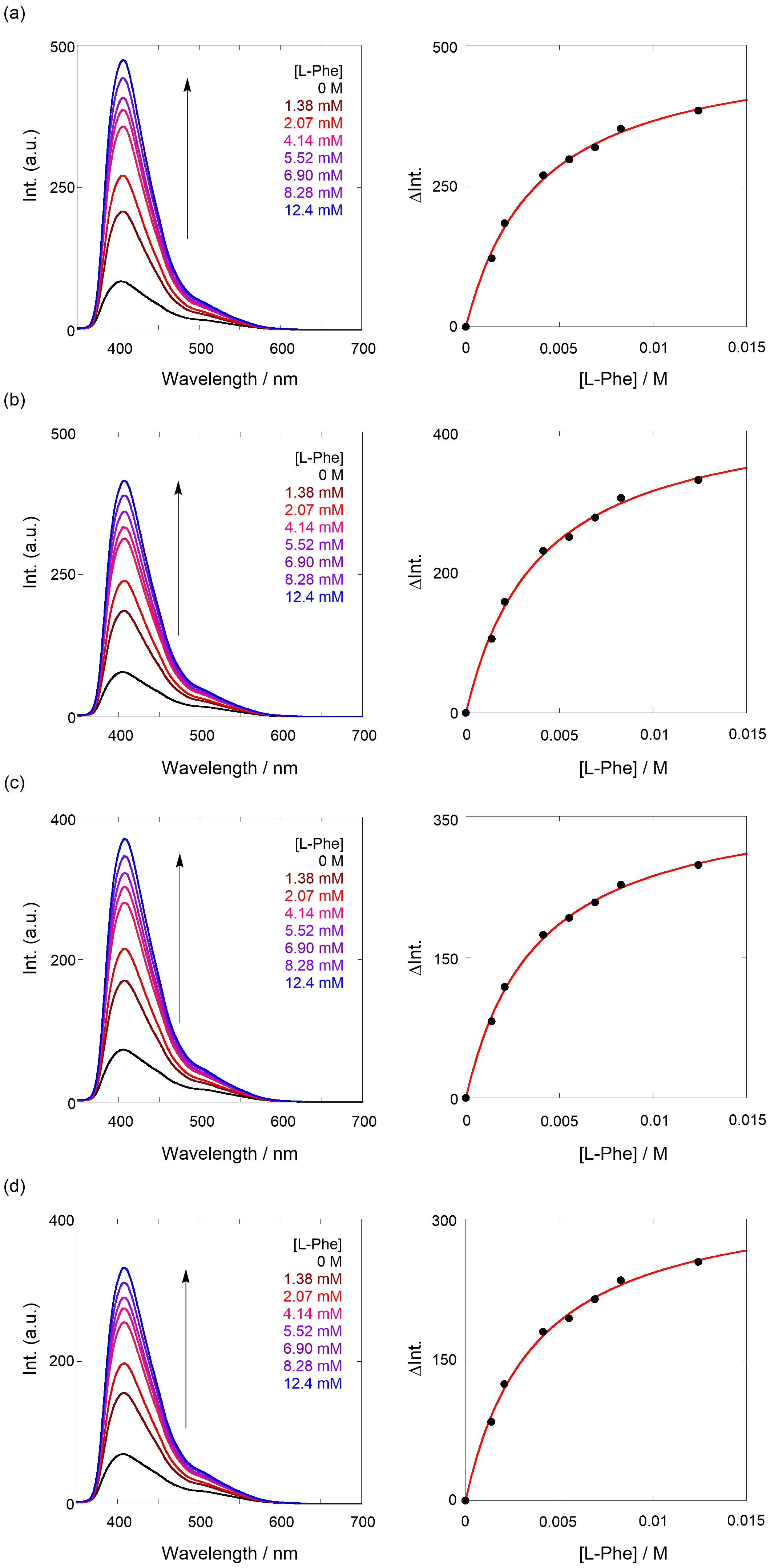


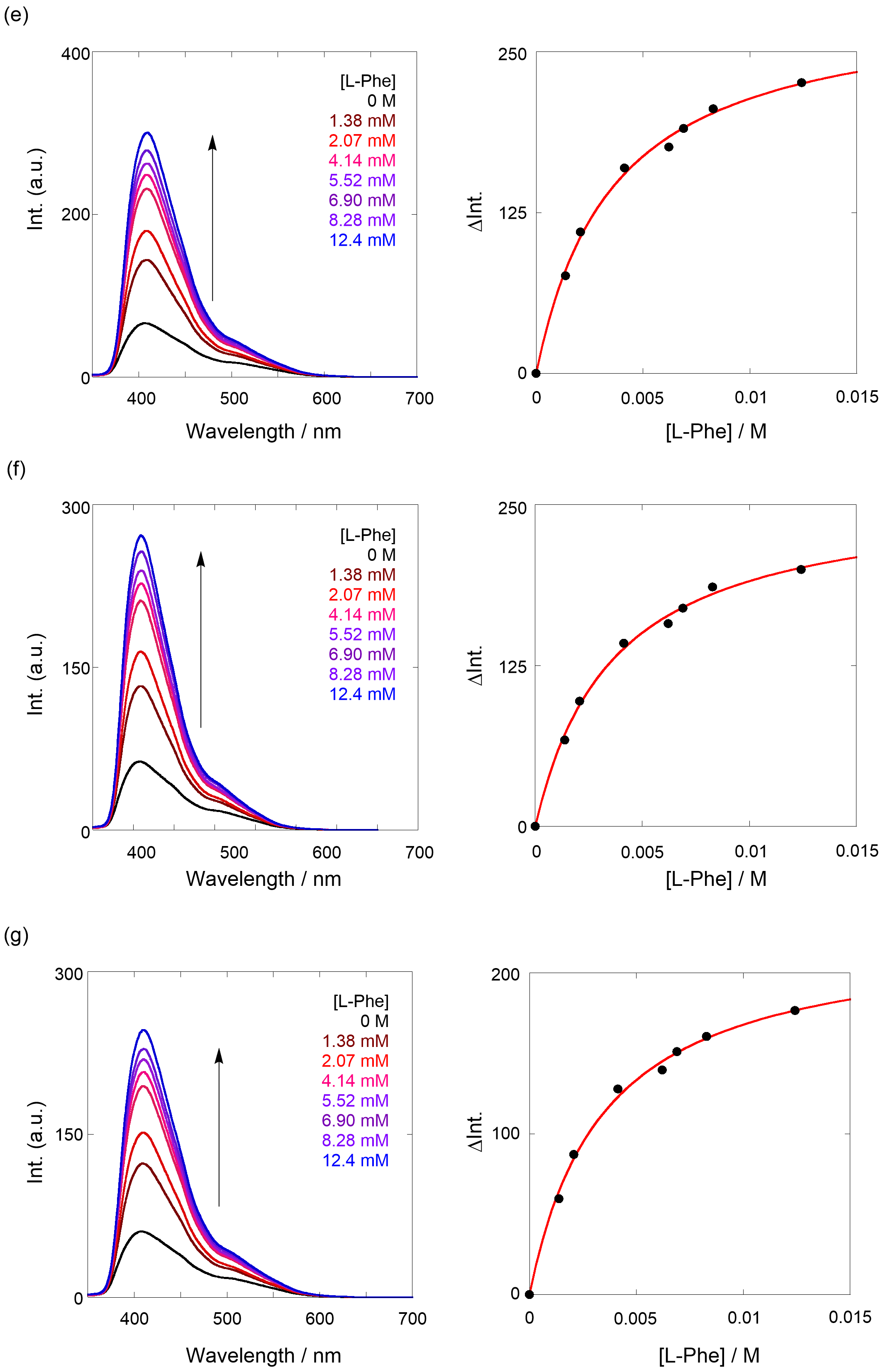


**Figure S6.** (*Left panels*) Fluorescence spectra (λ_ex_ 300 nm) of **1** (9.63 μM) upon the addition of L-Phenylalanine (0–12.4 mM, colored lines) in H_2_O at 25 °C, measured in a high-pressure cell. (*Right panels*) Nonlinear least-squares fitting, assuming 1:1 stoichiometry, to determine the binding constant (*K*) of L-phenylalanine with **1**. Pressure applied: (a) 40, (b) 80, (c) 120, (d) 160, (e) 200, (f) 240, (g) 280 MPa.


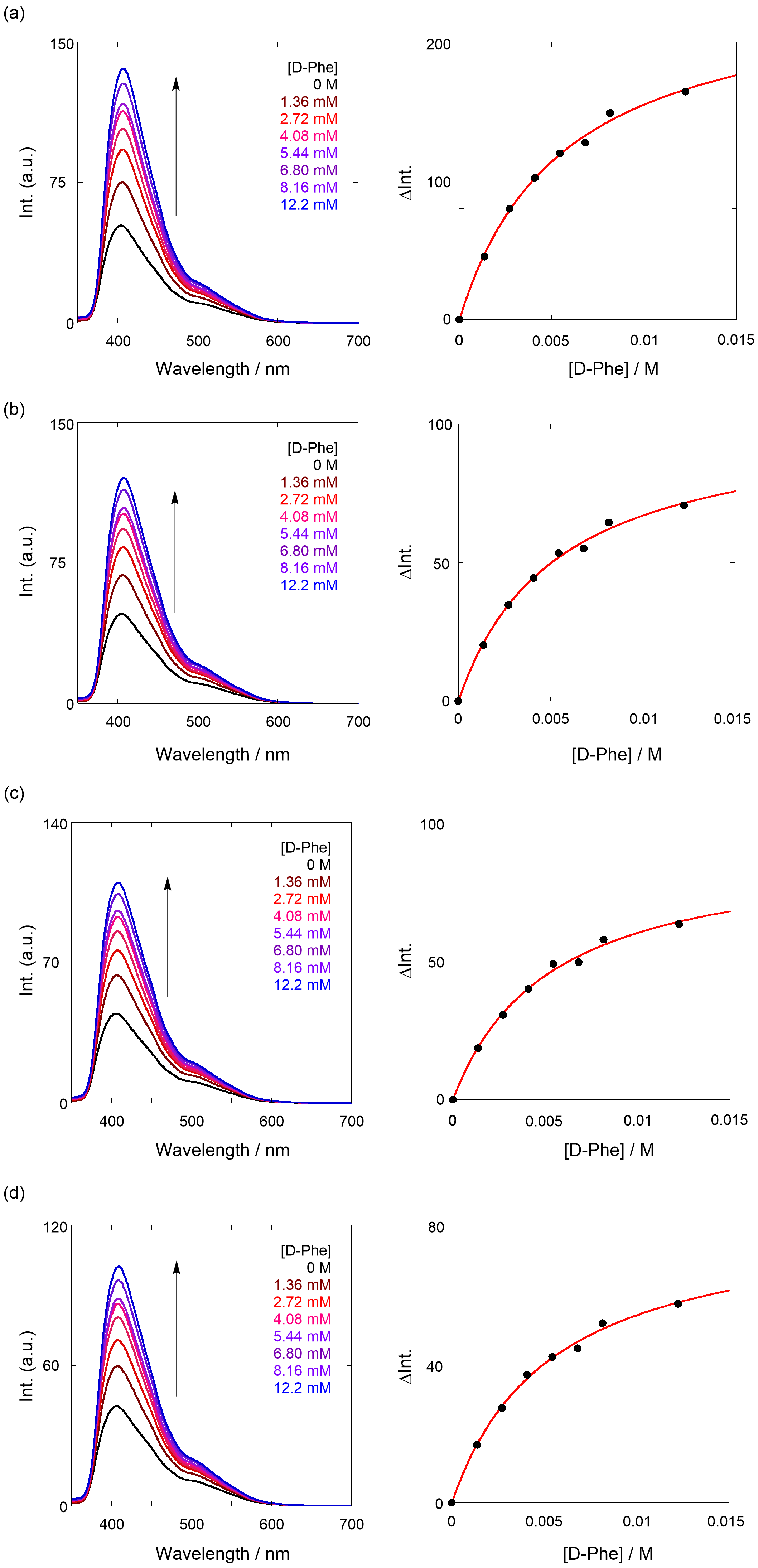


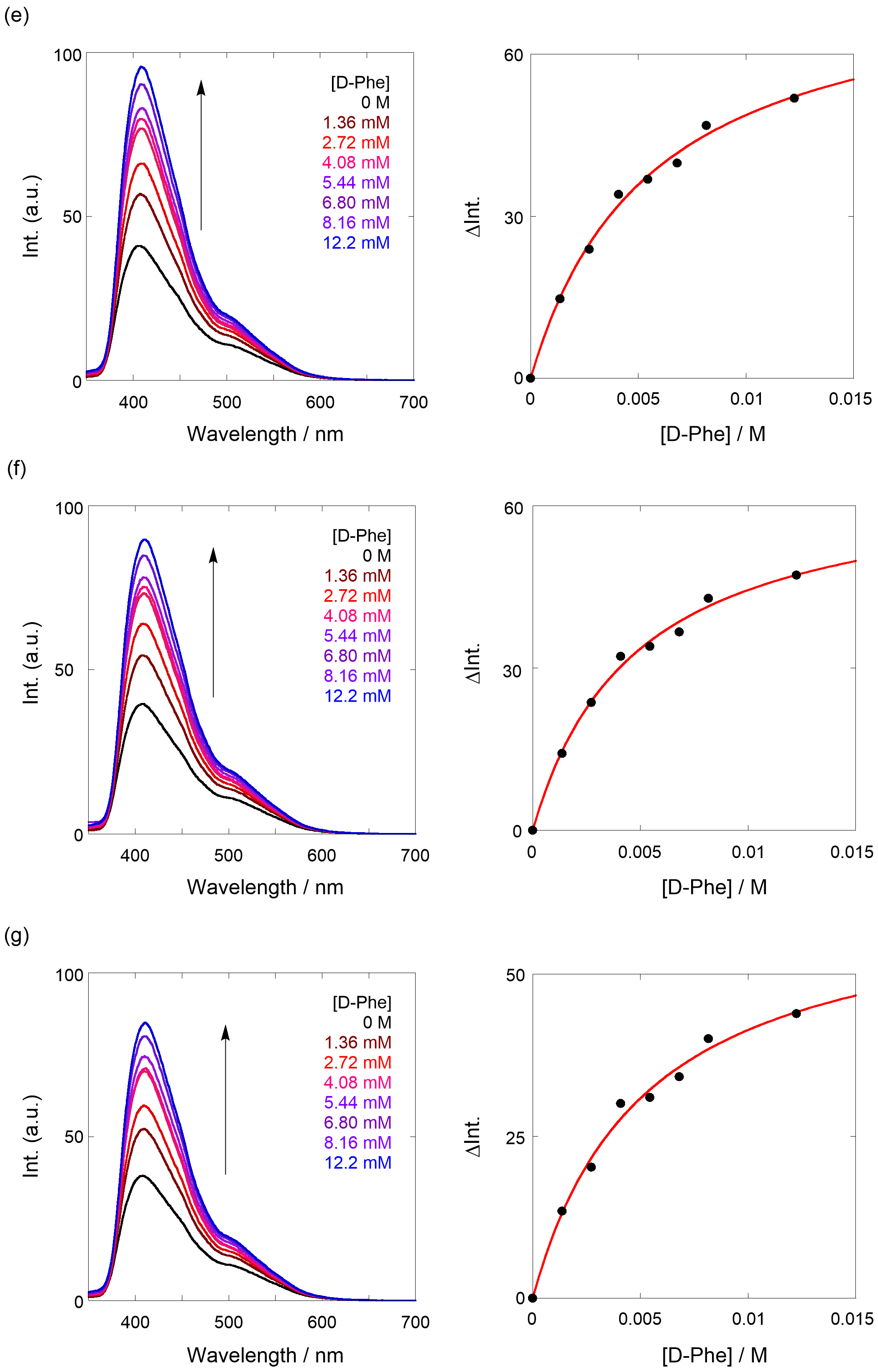


**Figure S7.** (*Left panels*) Fluorescence spectra (λ_ex_ 300 nm) of **1** (9.63 μM) upon the addition of D-Phenylalanine (0–12.2 mM, colored lines) in H_2_O at 25 °C, measured in a high-pressure cell. (*Right panels*) Nonlinear least-squares fitting, assuming 1:1 stoichiometry, to determine the binding constant (*K*) of D-phenylalanine with **1**. Pressure applied: (a) 40, (b) 80, (c) 120, (d) 160, (e) 200, (f) 240, (g) 280 MPa.


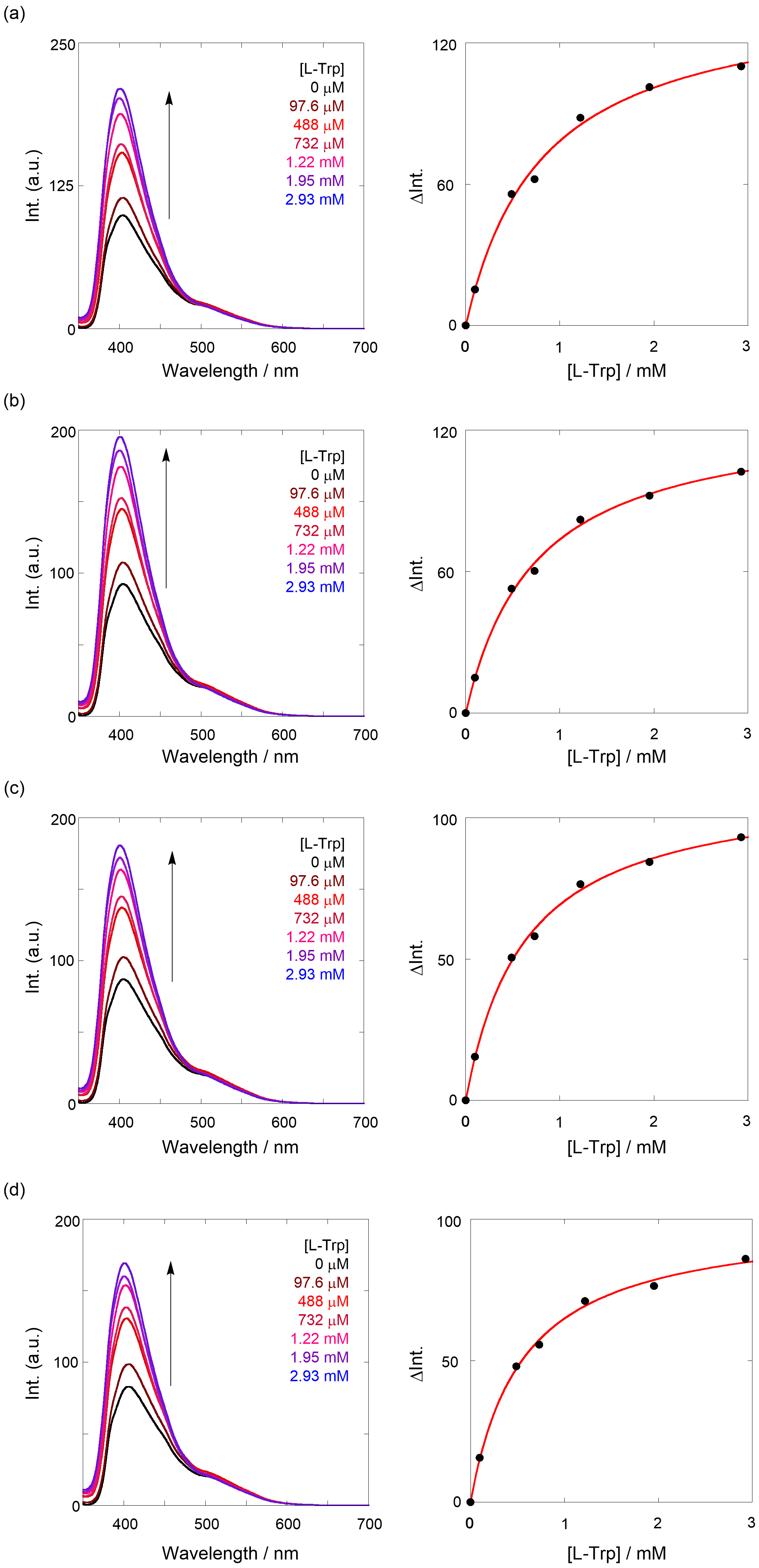


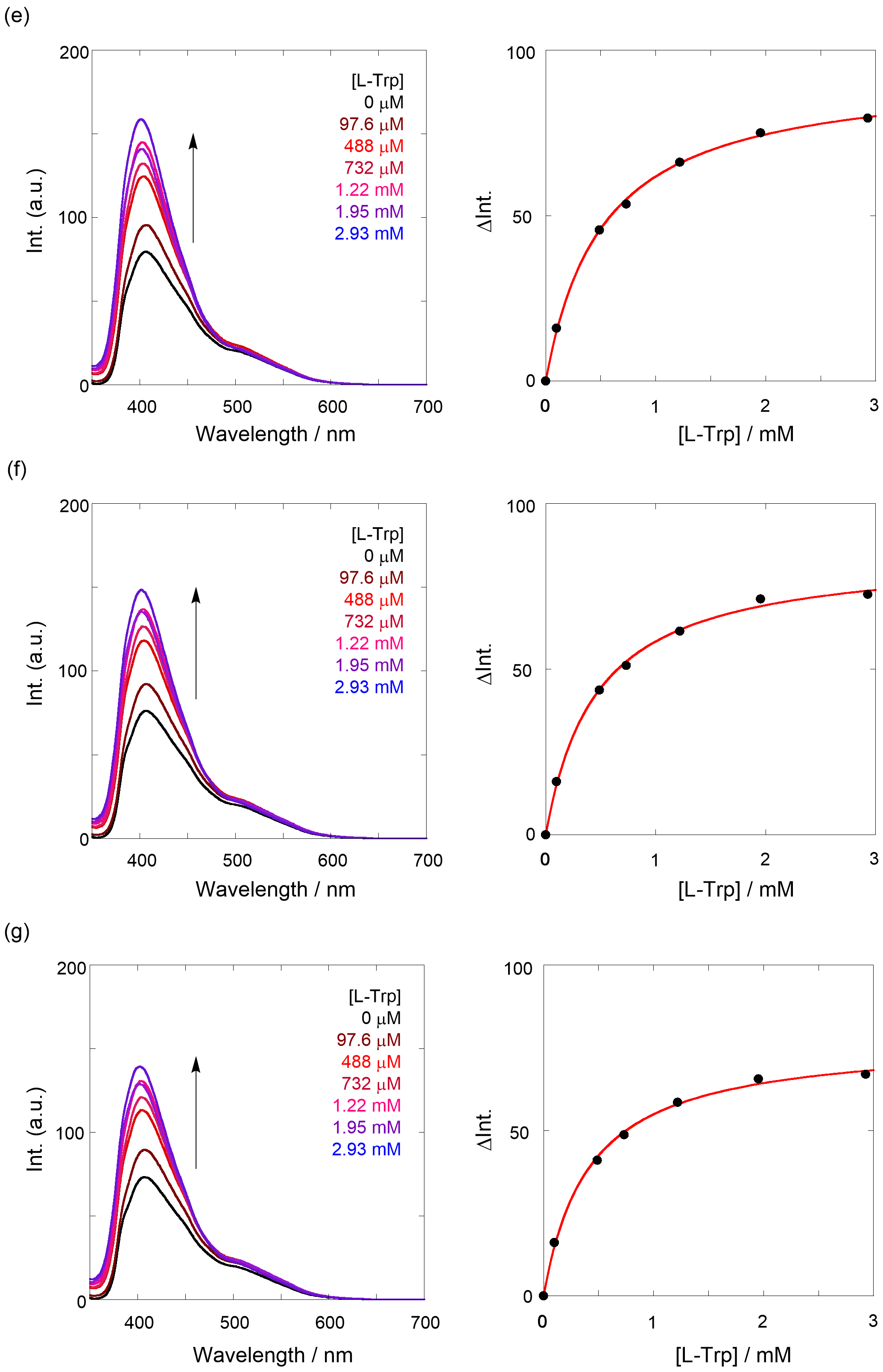


**Figure S8.** (*Left panels*) Fluorescence spectra (λ_ex_ 340 nm) of **1** (8.89 μM) upon the addition of L-Tryptophan (0–2.93 mM, colored lines) in H_2_O at 25 °C, measured in a high-pressure cell. (*Right panels*) Nonlinear least-squares fitting, assuming 1:1 stoichiometry, to determine the binding constant (*K*) of L-tryptophan with **1**. Pressure applied: (a) 40, (b) 80, (c) 120, (d) 160, (e) 200, (f) 240, (g) 280 MPa.


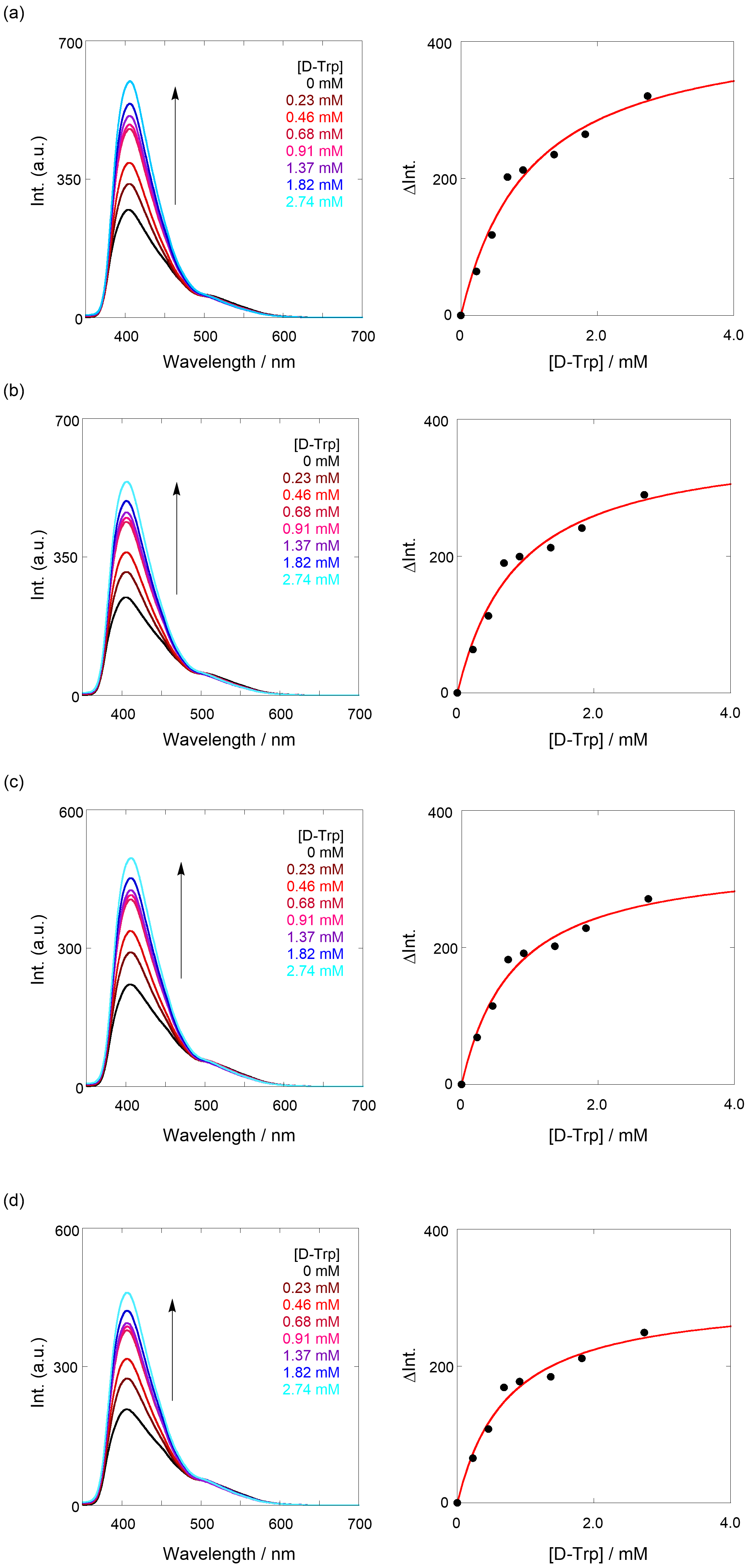


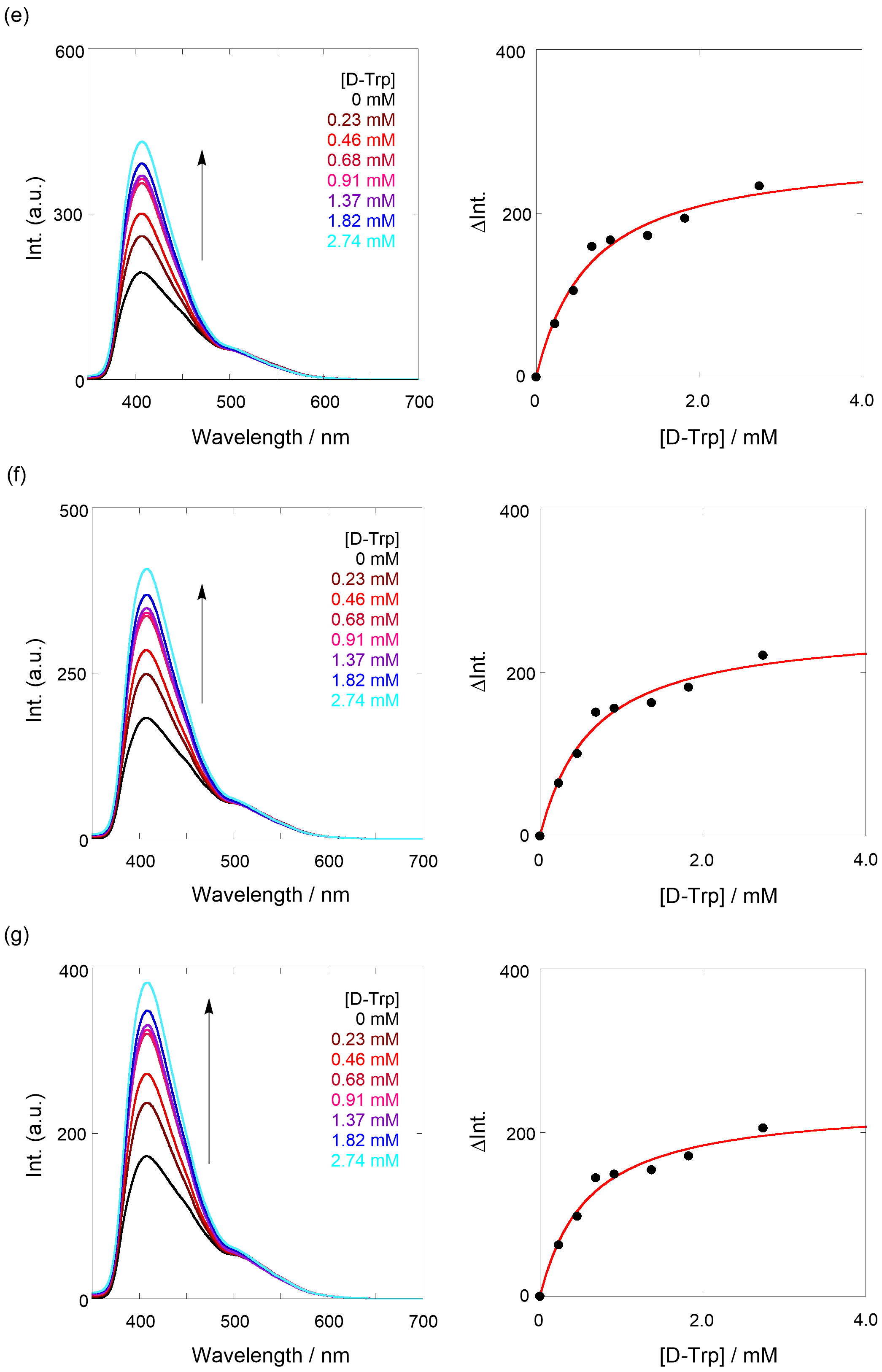


**Figure S9.** (*Left panels*) Fluorescence spectra (λ_ex_ 340 nm) of **1** (42.7 μM) upon the addition of D-Tryptophan (0–2.74 mM, colored lines) in H_2_O at 25 °C, measured in a high-pressure cell. (*Right panels*) Nonlinear least-squares fitting, assuming 1:1 stoichiometry, to determine the binding constant (*K*) of D-tryptophan with **1**. Pressure applied: (a) 40, (b) 80, (c) 120, (d) 160, (e) 200, (f) 240, (g) 280 MPa.


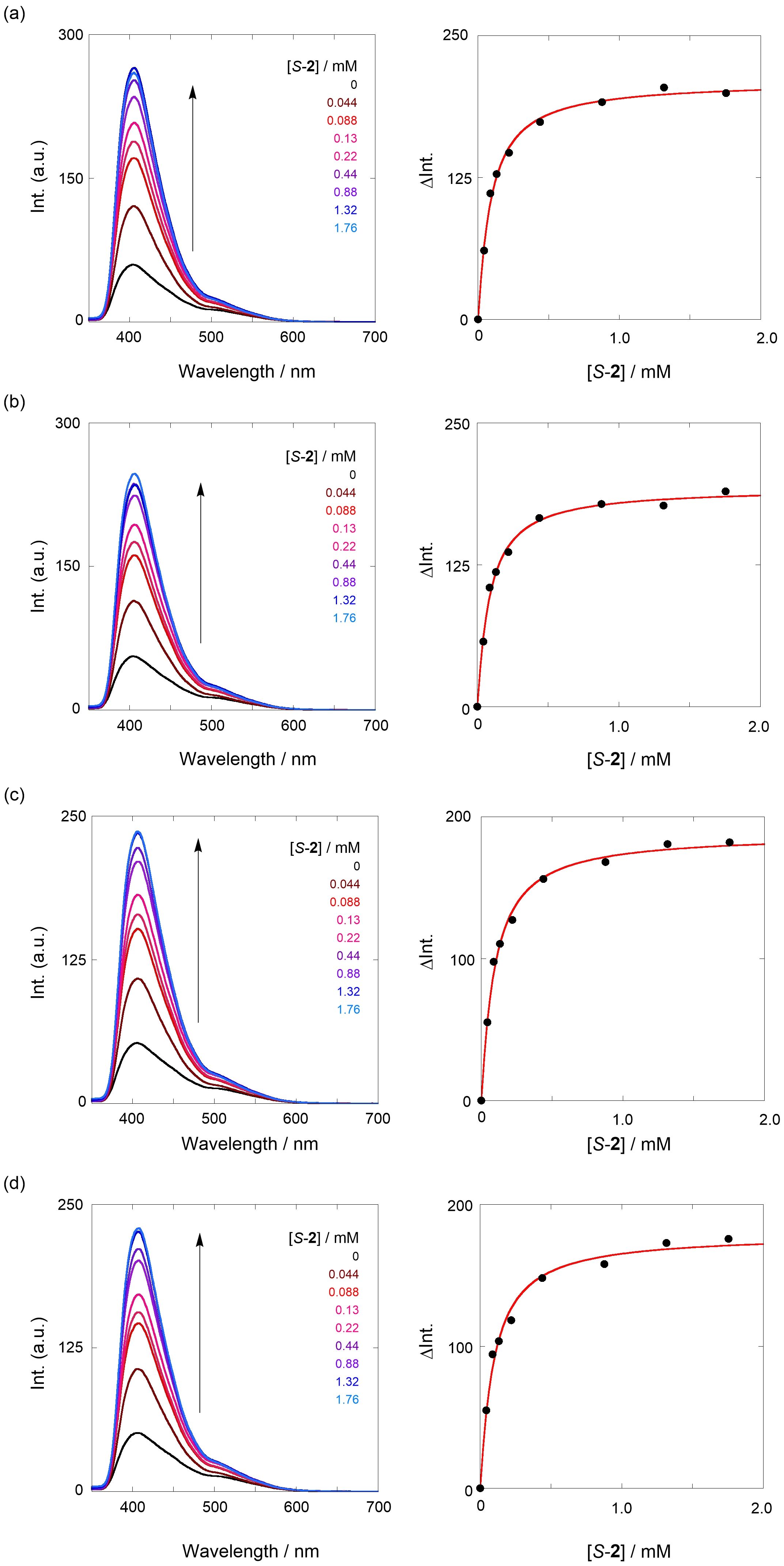


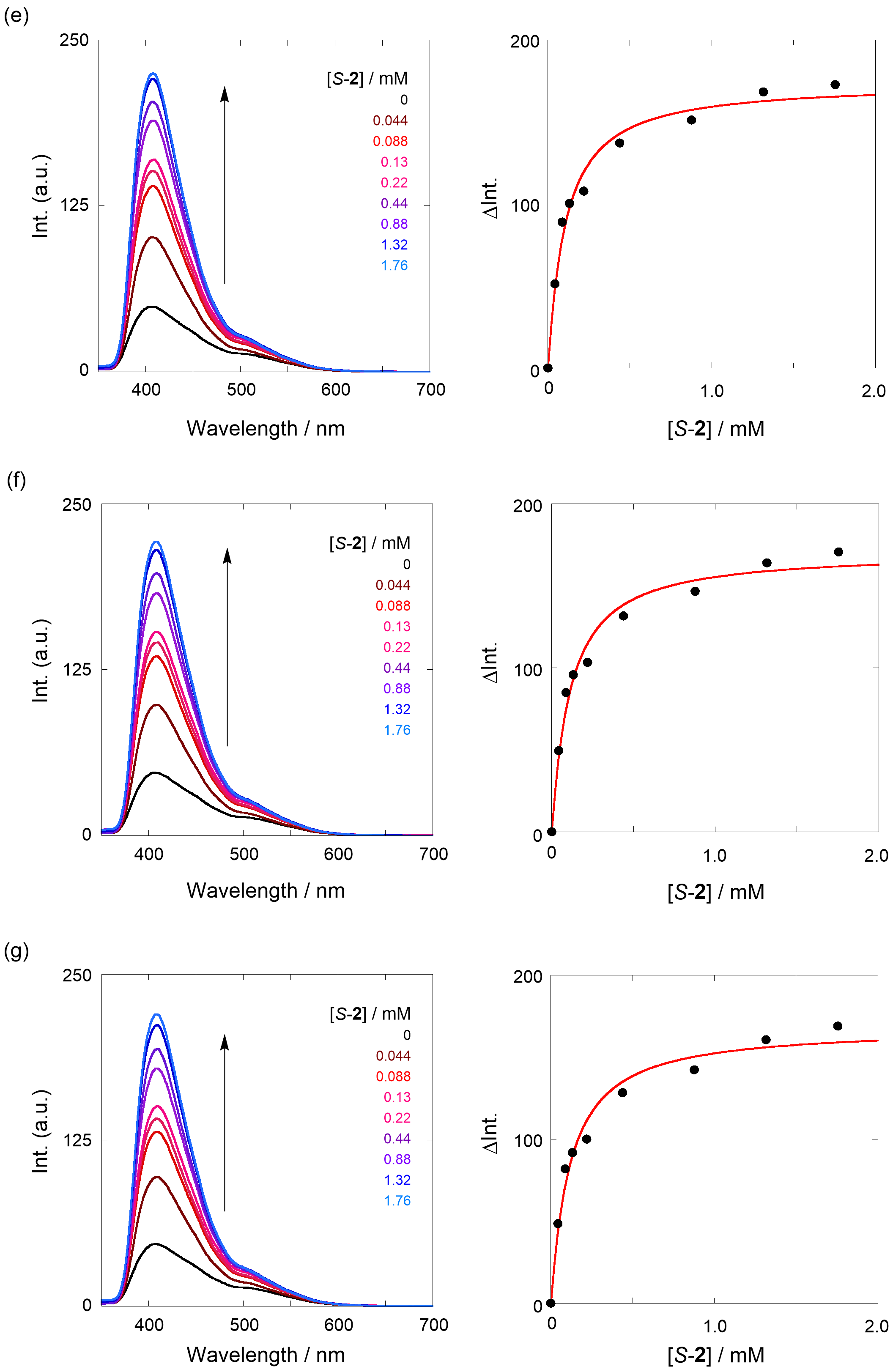


**Figure S10.** (*Left panels*) Fluorescence spectra (λ_ex_ 300 nm) of **1** (25.9 μM) upon the addition of *S*-**2** (0–1.76 mM, colored lines) in H_2_O at 25 °C, measured in a high-pressure cell. (*Right panels*) Nonlinear least-squares fitting, assuming 1:1 stoichiometry, to determine the binding constant (*K*) of *S*-**2** with **1**. Pressure applied: (a) 40, (b) 80, (c) 120, (d) 160, (e) 200, (f) 240, (g) 280 MPa.


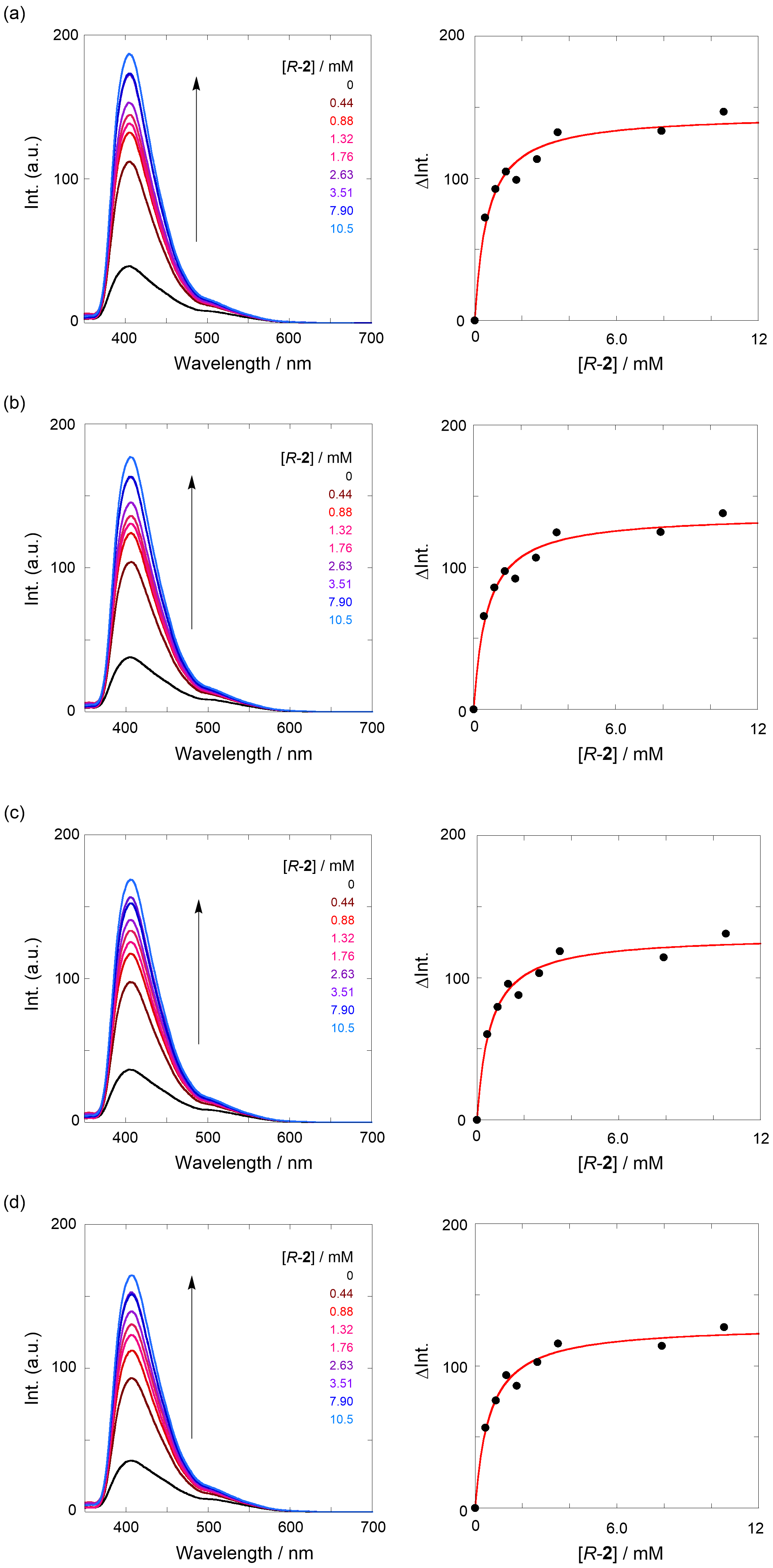


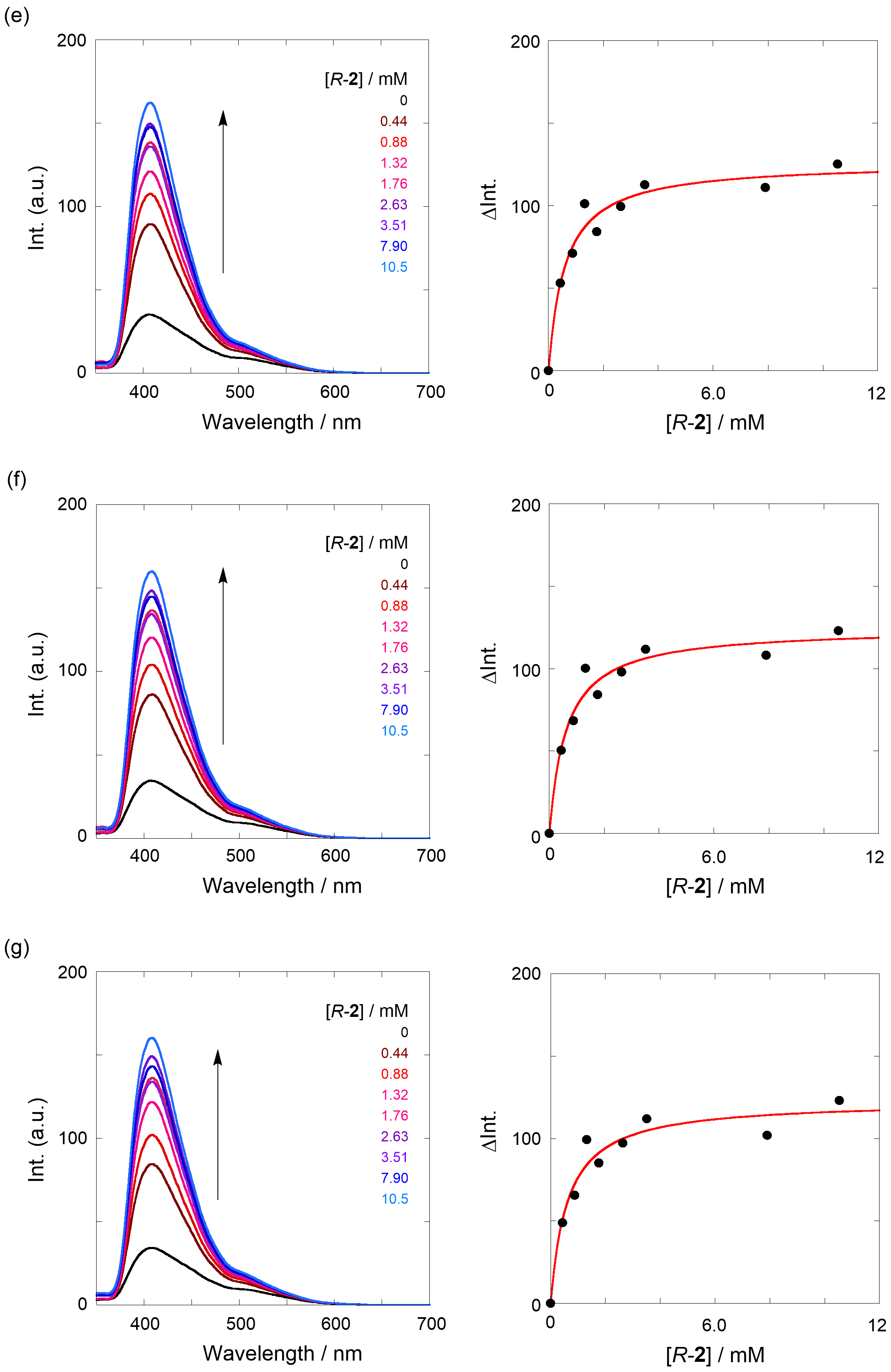


**Figure S11.** (*Left panels*) Fluorescence spectra (λ_ex_ 300 nm) of **1** (15.1 μM) upon the addition of *R*-**2** (0–10.5 mM, colored lines) in H_2_O at 25 °C, measured in a high-pressure cell. (*Right panels*) Nonlinear least-squares fitting, assuming 1:1 stoichiometry, was used to determine the binding constant (*K*) of *R*-**2** with **1**. Pressure applied: (a) 40, (b) 80, (c) 120, (d) 160, (e) 200, (f) 240, (g) 280 MPa.
